# The association between bacteria colonizing the upper respiratory tract and lower respiratory tract infection in young children: a systematic review and meta-analysis

**DOI:** 10.1016/j.cmi.2021.05.034

**Published:** 2021-09

**Authors:** Shantelle Claassen-Weitz, Katherine Y.L. Lim, Christopher Mullally, Heather J. Zar, Mark P. Nicol

**Affiliations:** 1) Division of Medical Microbiology, Department of Pathology, Faculty of Health Sciences, University of Cape Town, Cape Town, South Africa; 2) Marshall Centre for Infectious Diseases Research and Training, School of Biomedical Sciences, University of Western Australia, Perth, Australia; 3) Department of Paediatrics and Child Health, Red Cross War Memorial Children's Hospital, Cape Town, South Africa; 4) SAMRC Unit on Child & Adolescent Health, University of Cape Town, Cape Town, South Africa; 5) Institute of Infectious Disease and Molecular Medicine, Faculty of Health Sciences, University of Cape Town, Cape Town, South Africa; 6) Division of Infection and Immunity, School of Biomedical Sciences, University of Western Australia, Perth, Australia

**Keywords:** Aetiology, Bacteria, Children, Lower respiratory tract infection, Microbiome, Upper respiratory tract colonization

## Abstract

**Background:**

Bacteria colonizing the upper respiratory tract (URT) of young children play a key role in the pathogenesis of lower respiratory tract infection (LRTI).

**Objectives:**

To systematically review the literature on the association between bacteria colonizing the URT and LRTI among young children.

**Data sources:**

MEDLINE, Academic Search Premier, Africa-Wide Information and CINAHL, Scopus and Web of Science.

**Study eligibility criteria:**

Studies published between 1923 and 2020, investigating URT bacteria from LRTI cases and controls.

**Participants:**

Children under 5 years with and without acute LRTI.

**Methods:**

Three reviewers independently screened titles, abstracts and full texts. Meta-analysis was done using Mantel–Haenszel fixed- or random-effects models.

**Results:**

Most eligible studies (41/50) tested nasopharyngeal specimens when investigating URT bacteria. Most studies were of cross-sectional design (44/50). Twenty-four studies were performed in children in lower- or lower-middle-income countries (LMICs). There was higher prevalence of *Haemophilus influenzae* (pooled OR 1.60; 95% CI 1.23–2.07) and *Klebsiella* spp. (pooled OR 2.04; 95% CI 1.17–3.55) from URT specimens of cases versus controls. We observed a positive association between the detection of *Streptococcus pneumoniae* from URT specimens and LRTI after excluding studies where there was more antibiotic treatment prior to sampling in cases vs. controls (pooled OR 1.41; 95% CI 1.04–1.90). High density colonization with *S. pneumoniae* (>6.9 log^10^ copies/mL) was associated with an increased risk for LRTI. The associations between both *Streptococcus* and *Haemophilus* URT detection and LRTI were supported, at genus level, by 16S rRNA sequencing. Evidence for the role of *Moraxella catarrhalis* and *Staphylococcus aureus* was inconclusive.

**Conclusions:**

Detection of *H. influenzae* or *Klebsiella* spp. in the URT was associated with LRTI, while evidence for association with *S. pneumoniae* was less conclusive. Longitudinal studies assessing URT microbial communities, together with environmental and host factors are needed to better understand pathogenesis of childhood LRTI.

## Introduction

Over the past two decades, widespread introduction of conjugate vaccines against *Streptococcus pneumoniae* and *Haemophilus influenzae* type b (Hib) has contributed to a global reduction in the incidence and severity of lower respiratory tract infection (LRTI) [1,2]. Yet LRTI remains the leading cause of morbidity and mortality among children under 5 years, causing 808 920 deaths in 2017 [3]. The Global Burden of Disease Study 2017 reported pneumococcal pneumonia as the leading cause of LRTI deaths [2,4].

**Fig. 1:**
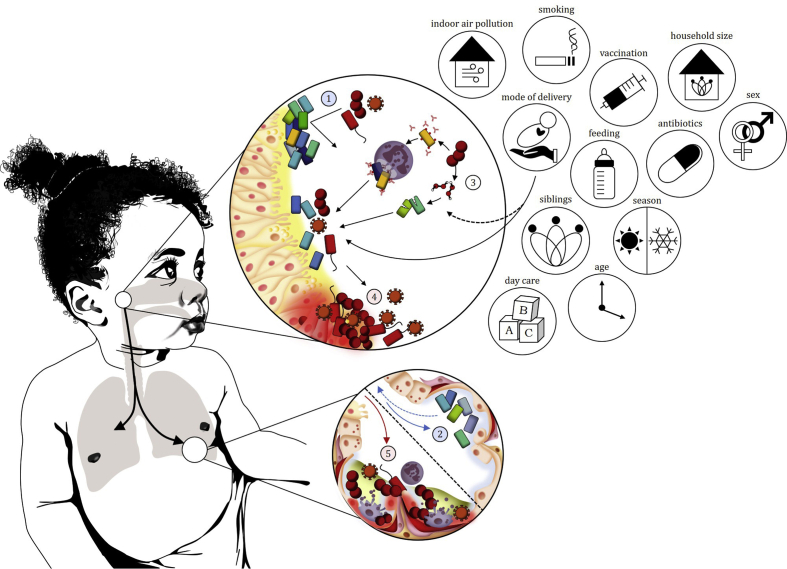
Imbalances in upper respiratory tract (URT) bacterial communities may result in translocation of dysbiotic bacterial communities to the lower respiratory tract (LRT), causing infection. (1) Commensal bacteria with low pathogenic potential confer colonization resistance against potential pathogens. (2) Bacterial communities from the URT translocate to the lungs where they are detected as stable resident or transient LRT communities. (3) Bacterial–bacterial interactions in the URT may be competitive or synergistic allowing potential pathogens to colonize. Environmental exposures may influence bacterial–bacterial and bacterial–host interactions, or directly impact on select bacteria within the community. (4) Perturbations of the bacterial community (dysbiosis), resulting from pathogen exposure, viral-bacterial, bacterial-bacterial interactions and environmental risk factors, may result in inflammation and damage to the URT epithelium. (5) Dysbiotic bacterial communities translocate to the LRT and cause inflammation and damage to the respiratory epithelium.

The conventional model of LRTI pathogenesis has been challenged with the advent of sequence-based methods for characterizing microbial communities. The concept that a pathogen from the upper respiratory tract (URT) invades and infects sterile lungs has been replaced by the notion that the lungs harbour transient or persistent bacterial communities derived from the URT. Shifts in URT bacterial communities may shape bacterial communities in the lungs associated with health or disease (Fig. 1) [[5], [6], [7]].

However, studies of the association between URT bacterial communities and LRTI in children have shown varying results. We therefore systematically reviewed data from studies investigating the association between URT bacterial prevalence and LRTI in children under the age of five years.

## Materials and methods

A comprehensive description of the methodology is provided in Appendix 1. The review protocol (CRD42020200544) is available from the International Prospective Register of Systematic Reviews (PROSPERO) [8].

We followed the Preferred Reporting Items for Systematic Reviews and Meta-Analyses (PRISMA) criteria for reporting of systematic reviews [9] (Appendix 1). We searched six databases for articles reporting on the detection of URT bacteria in children with LRTI and controls (Appendix 1; Table 1). Definitions used are outlined in Appendix 1.

**Table 1:** Eligibility criteria

PICOS strategy	Inclusion criteria (all needed)	Exclusion criteria (any)
Population	• Children ≤5 years of age • Children with acute LRTI • Children providing URT specimens • Children enrolled from hospital (inpatient or outpatient), primary healthcare clinic or community settings	• Children >5 years of age • Children with cystic fibrosis • Children with RTI (infection of the respiratory tract without information on the site of infection) • Children with URTI where no LRTI symptoms were reported • Only a subset of children was diagnosed with acute LRTI, but no disaggregated analyses were done • Children provided LRT but not URT specimens
Intervention	• Not applicable	• Not applicable
Comparator	• Children ≤5 years of age • Children with or without URTI	• Children >5 years of age • Children with RTI (if not clearly defined as URTI or non-severe LRTI) • Children with LRTI were included only if: (1) the population was children with severe LRTI and (2) the comparator group included children with non-severe LRTI accounting for <5% of the total comparator group
Outcome	• Bacterial ± viral ± fungal prevalence data from URT specimens from both population and comparator groups	• Viral/fungal prevalence data only
Study design	• Cross-sectional and longitudinal case-control studies	• Interventional studies (vaccination or antibiotic) • Outbreak investigations • Animal studies • Non-primary literature (including reviews, dissertations, editorials, protocol studies and clinical guidelines) • No access to abstract and/or full-text

PICOS, patients, intervention, comparator, outcomes, study design; (L)/(U)RTI, (lower)/(upper) respiratory tract infection.

We extracted data from eligible studies using a predefined data extraction template (Appendix 1) and evaluated eligible studies using the Newcastle–Ottawa Quality Assessment Scale (NOS) for case–control studies [10].

We determined the association between the prevalence of URT bacterial species and the risk of LRTI using pooled odds ratios (ORs) and corresponding confidence intervals (CIs). Pooled ORs were calculated in RevMan [11] using Mantel Haenszel fixed- or random-effects models based on between-study heterogeneity. We excluded studies with potentially overlapping participants from all quantitative analyses to avoid “double counting” aetiological data from a single set of participants.

## Results

### Study selection and characteristics of the included studies

We identified 6432 studies for title and abstract screening (Appendix 2). A total of 6309 studies were excluded based on titles and abstracts screened. Of the 6309 studies, 15 full texts could not be assessed for eligibility due to translational issues (Appendix 2). Of the 123 studies identified for full-text review, 73 were identified as ineligible (Appendix 2). In total, 50 studies were included in the systematic review (The reference list of eligible studies included in the review is provided in Appendix 2).

**Fig. 2:**
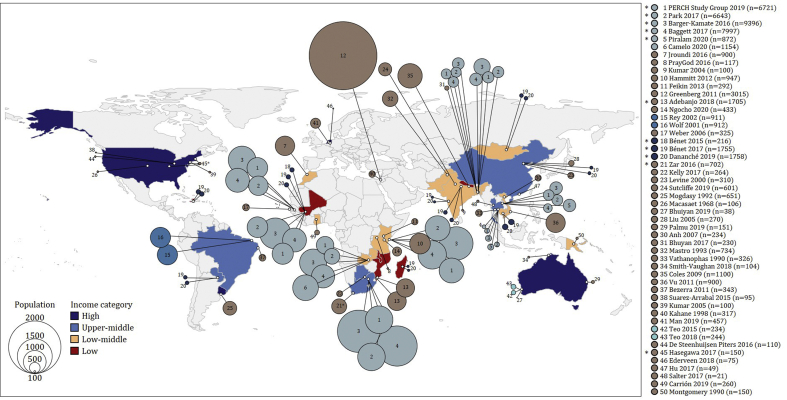
Eligible studies included in this review (*N* = 50) were performed across 29 countries. The 50 eligible studies are listed on the right of the figure. The reference list of eligible studies included in the review is provided in Appendix 2. Multicentre studies are denoted with an asterisk. Studies shown in the same shade of blue represent potential overlap in participants across these studies.

The characteristics of the 50 eligible studies, published between June 1968 and June 2020, are summarised in Appendix 2. Forty single-centre and ten multicentre studies were identified across 29 countries (high-income (five countries), upper-middle-income (six countries), lower-middle-income (12 countries) and low-income (six countries)) (Fig. 2; Appendix 2). Most participants were from low-income (*n* = 12 954/55 495, 23%) or lower-middle-income (*n* = 21 764/55 495, 39%) countries (Appendix 2).

Five studies followed children longitudinally, whilst 44 used a cross-sectional case–control design (Appendix 2). One [study 34] included cross-sectional and longitudinal components.

URT specimens tested included nasopharyngeal swabs (25 studies), nasopharyngeal aspirates (five studies), nasal swabs (five studies), throat swabs (two studies) or oropharyngeal swabs (one study) (Appendix 2). Seven studies by the Pneumonia Etiology Research for Child Health (PERCH) Group [studies 1-6] and Feikin and colleagues [study 11] used a combined nasopharyngeal and oropharyngeal swab, while a further five studies tested two different specimens. Overall, 82% of studies included nasopharyngeal specimens (swabs, aspirates, or combined specimens).

Most studies (70%) screened for more than one bacterium using culture (*n* = 10), PCR (*n* = 13), culture and PCR (*n* = 3) or short fragment sequencing of 16S rRNA gene amplicons (*n* = 9) (Appendix 2). The nine 16S rRNA profiling studies were carried out in high-income (*n* = 6) and upper-middle-income (*n* = 3) countries.

The 50 eligible studies primarily enrolled cases from hospital (*n* = 28), ambulatory (*n* = 8) and community (*n* = 7) settings, while controls were primarily enrolled from community (*n* = 17), ambulatory (*n* = 16) and hospital (*n* = 6) settings (Appendix 2).

Reporting of antibiotic treatment prior to sampling was variable (Appendix 2). Half of eligible studies did not provide vaccination details (*n* = 25) (Appendix 2). Nineteen studies reported on pneumococcal conjugate vaccine (PCV). Other vaccinations included Hib (*n* = 9); diphtheria, tetanus, and pertussis (DTP/DTaP) (*n* = 4); measles (*n* = 3) and influenza (*n* = 2).

Studies primarily included participants up to 5 years (68%), 2 years (14%) or 1 year of age (12%) (Appendix 2). More males were included as cases compared with controls in 40% of studies, while more females were included as cases compared with controls in 18% of studies. Fifteen studies did not report on sex. Studies adjusted for age (*n* = 27), season (*n* = 15), sex (*n* = 4), site (*n* = 4), HIV status (*n* = 1) and nutritional status (*n* = 1), while 21 studies did not provide adjustments for potential confounders.

Overall, 72% (36/50) studies were considered to have low risk of bias (NOS ≥7) (Appendix 2).

### Associations between URT bacteria and LRTI

Seven studies including potentially overlapping participants were excluded to avoid “double counting” (Fig. 2; Appendix 2), and one further study was excluded as no prevalence data were provided. Data from 42 studies were available for meta-analysis (Appendix 2).

**Fig. 3:**
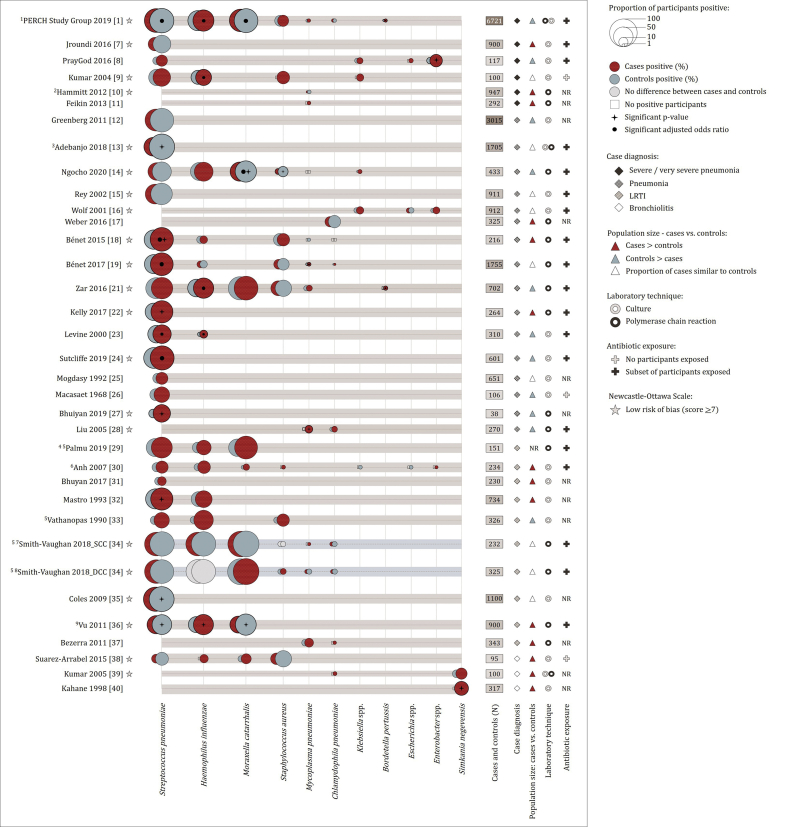
Proportion of lower respiratory tract infection (LRTI) cases and controls from which bacteria were detected in the upper respiratory tract (URT) using culture or PCR. Only bacteria for which two or more eligible studies provided prevalence data are represented. Proportions of cases/controls from which each of the respective bacteria were detected are represented by bubbles of different sizes. Proportions were calculated using the number of participants positive divided by the number of participants tested. The group (cases or controls) with a higher proportion of participants positive is plotted in front of the group with a lower proportion of participants positive (Red bubbles: cases; Blue bubbles: controls). Grey bubbles: no differences between case control groups. Unfilled squares: zero prevalence. The reference list of eligible studies included in the review is provided in Appendix 2. ^1^Data from chest radiography positive cases. ^2^Data excludes controls with respiratory tract infection. ^3^Results from culture not PCR. PCR was only performed for culture negative cases (not controls). Antibiotic exposure among cases was higher compared to controls (97% vs. 27%). ^4^Control specimens include specimens collected asymptomatically or during URTI/acute otitis media episodes. ^5^Data from specimens screened, not participants. ^6^Data from cases represent both pneumonia and acute bronchiolitis groups. ^7^SCC, Same Child Control cohort (LRTI specimen matched to non-LRTI specimen from the same child). ^8^DCC, Different Child Control cohort (LRTI specimen matched to non-LRTI specimen from a different child). ^9^Data from cases represent both pneumonia and other LRTI groups.

Fig. 3 represents URT bacterial prevalence from culture or PCR, for bacteria which were reported in at least two studies (34 studies). Among these, participants were most frequently screened for *S. pneumoniae* (*n* = 26), *H. influenzae* (*n* = 14), *M. catarrhalis* (*n* = 8), *S. aureus* (*n* = 10), *Mycoplasma pneumoniae* (*n* = 10) and *Chlamydophila pneumoniae* (*n* = 8).

**Fig. 4:**
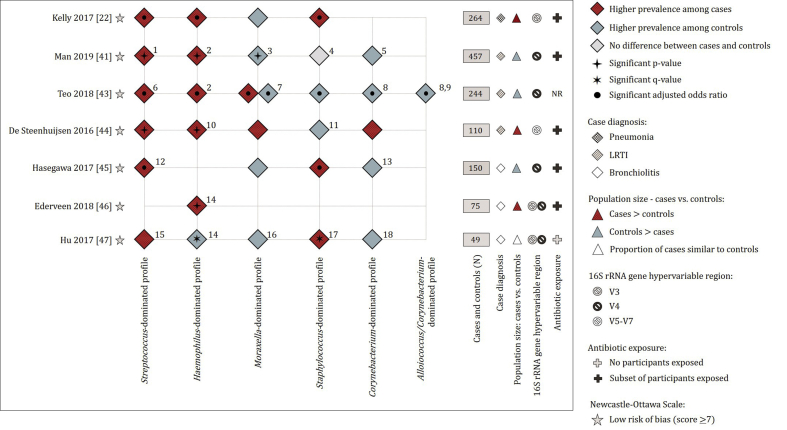
Bacterial profiles from 16S rRNA gene amplicon sequencing of upper respiratory tract samples from LRTI cases and controls. Red diamonds: microbiota profile group/genus was more prevalent among cases compared to controls. Blue diamonds: microbiota profile group/genus was more prevalent among controls compared to cases. Grey diamonds: no differences in the prevalence of microbiota profile group/genus among cases and controls. The reference list of eligible studies included in the review is provided in Appendix 2. (1) *Streptococcus pneumoniae*-dominated profile. (2) *Haemophilus influenzae*/*haemolyticus*-dominated profile. (3) *Moraxella catarrhalis*/*nonliquefaciens*-dominated profile. (4) *Staphylococcus aureus*/*epidermidis*-dominated profile. (5) *Corynebacterium propinquum*/*Dolosigranulum pigrum*-dominated profile. (6) *Streptococcus pneumoniae*/*pseudopneumoniae*-dominated profile. (7) *Moraxella catarrhalis*/*lincolnii*-dominated profile. (8) *Corynebacterium propinquum*/*pseudodiphtheriticum*-dominated profile. (9) *Alloiococcus otidis* (*Dolosigranulum pigrum*)-dominated profile. (10) *Haemophilus influenzae*-dominated profile. (11) *Staphylococcus aureus*-dominated profile. (12) Mixed profile (*Streptococcus*/*Tumebacillus*-dominated profile). (13) *Corynebacterium*/*Dolosigranulum*-dominant profile. (14) *Haemophilus* genus. (15) *Streptococcus* genus. (16) *Moraxella* genus. (17) *Staphylococcus* genus. (18) *Corynebacterium* genus.

Five of eight studies using 16S rRNA sequencing designated specimens into “microbiota profile groups” based on the genus detected at the highest relative abundance from each of the specimens. Case–control prevalence data were primarily provided for *Streptococcus*-, *Haemophilus*-, *Moraxella*-, *Staphylococcus*-, *Corynebacterium*- and *Alloiococcus/Corynebacterium*-dominated profiles (microbiota profile groups) (Fig. 4). Two [studies 46 and 47] reported relative abundances of bacterial genera from cases and controls (Fig. 4). One study, excluded from Fig. 4, longitudinally investigated microbiota profiling groups between healthy and LRTI states [study 48]. No distinct microbiota profiles were reported for healthy and LRTI states within this cohort, however, the study reported that respiratory illness coincided with a perturbation in bacterial community profiles which were unique for each child and each illness episode [study 48].

#### Streptococcus pneumoniae

The majority (16/26, 62%) of studies using culture or PCR showed a higher prevalence of *S. pneumoniae* among cases compared to controls (Fig. 3); however, the pooled OR did not show a significant association (pooled OR 1.16; 95% CI 0.86–1.57) (Appendix 3: Fig. S1). A sensitivity analysis, including data from 13 studies with NOS scores ≥7 and > 200 participants, showed a higher prevalence among controls than cases (pooled OR 0.93; 95% CI 0.60–1.43) (Appendix 3: Fig. S2). Subgroup analysis of eight studies where antibiotic treatment prior to sampling was more prevalent among cases than controls showed no significant difference in prevalence of *S. pneumoniae* among these groups (pooled OR 0.75; 95% CI 0.43–1.31) (Appendix 3: Table S1). Following the exclusion of these eight studies, a significant association was observed between *S. pneumoniae* and LRTI (*n* = 18) (pooled OR 1.41; 95% CI 1.04–1.90).

The PERCH Study [study 1], which investigated aetiology in settings with high PCV coverage, reported a significantly higher prevalence of *S. pneumoniae* among controls compared to hospitalised LRTI cases (Fig. 3; Appendix 3: Fig. S1,S2). After applying a pathogen-specific PCR density threshold (>6.9 log^10^ copies/mL), PERCH reported that *S. pneumoniae* was associated with an increased risk for LRTI. Another study reported a significantly higher median density of *S. pneumoniae* from participants with radiologically confirmed pneumonia compared to healthy controls (7.8 × 10^6^ copies/mL vs. 7.9 × 10^5^ copies/mL; p < 0.0001) [study 36].

Eight of twelve studies reporting on pneumococcal serotypes showed higher prevalence of vaccine type pneumococci from cases compared to controls (Appendix 4). Of these, two studies reported higher PCV vaccination from controls [studies 7 and 13], while two studies reported no PCV vaccination of either cases and controls [studies 12 and 18]. Five of the eight studies provided no details on PCV status of individual participants [studies 15,32,36 and 50], two of which reported a higher prevalence of non-vaccine pneumococcal serotypes from controls compared with cases [studies 32 and 36] (Appendix 4). Longitudinal analysis showed that pneumococcal serotypes 1, 5, 7, 9, 10, 14, 18, 45 and 46, classified as invasive serotypes [study 50], were more frequently detected during LRTI episodes compared to controls [study 50].

All six studies performing 16S rRNA gene amplicon sequencing reported higher prevalence of *Streptococcus*-dominated profiles or higher relative abundance of *Streptococcus* genus among cases than controls (Fig. 4), with statistically significant findings in five studies.

#### Haemophilus influenzae

*Haemophilus influenzae* was more prevalent among cases than among controls in 86% (12/14) of studies using culture or PCR (Fig. 3) (pooled OR 1.60; 95% CI 1.23–2.07)] (Appendix 3: Fig. S3). Sensitivity analysis including studies with NOS scores ≥7 and > 200 participants (seven studies) showed similar findings (pooled OR 1.24; 95% CI 0.96–1.62)] (Appendix 3: Fig. S4), as did subgroup analysis (Appendix 3: Table S2).

Only five of the 14 studies performed *H. influenzae* serotyping [studies 1,14,21,23 and 32], three of which provided non-typeable *H. influenzae* (NTHI), non-type b *H. influenzae* (non-type b) and/or Hib prevalence data. NTHI prevalence was reported at 91.3% from cases and 96.4% from controls [study 32]. Non-type b *H. influenzae* prevalence was higher among cases than in controls (55.4% vs. 49.6%) (OR 1.22; 95% CI 1.07–1.40) [study 1]. Hib prevalence was low overall, but higher among cases than controls (2.4% vs. 1.9%) (OR 1.74; 95% CI 1.14–2.66) [study 1]): and (7.3% vs. 1.9%) (OR 4.12; 95% CI 1.01–16.80) [study 23]. Hib vaccine coverage was reported by 7/14 studies [studies 1,14,18,19,21,23,30], of which two reported no Hib vaccination [studies 23 and 30] while three reported higher coverage of Hib vaccination in controls [studies 1,14 and 19].

Five of six 16S rRNA gene amplicon sequencing studies reported significantly higher prevalence of *Haemophilus*-dominated profiles or higher relative abundance of *Haemophilus* genus from cases compared to controls (Fig. 4).

#### Moraxella catarrhalis

Four culture or PCR studies reported higher prevalence of *M. catarrhalis* from cases, whilst four studies reported higher prevalence among controls (Fig. 3) (pooled OR 1.09; 95% CI 0.55–2.15; Appendix 3: Fig. S5). *Moraxella catarrhalis* prevalence was borderline lower among cases when including studies with NOS scores ≥7 and > 200 participants (five studies) (pooled OR 0.71; 95% CI 0.51–1.00) (Appendix 3: Fig. S6). Studies including participants under 5 years, and those where more cases had antibiotic treatment prior to sampling than controls, showed significantly higher prevalence of *M. catarrhalis* among controls than among cases (Appendix 3: Table S3).

One study showed that *M. catarrhalis* URT density was significantly higher among LRTI cases when compared to healthy controls (p < 0.0001) [study 36], while another study reported higher bacterial densities among controls [study 21].

One 16S rRNA gene amplicon sequencing study reported higher prevalence of *Moraxella*-dominated profiles from cases, whilst four reported higher prevalence of *Moraxella*-dominated profiles or higher relative abundance of *Moraxella* genus from cases among controls (Fig. 4). One study [study 43] investigated profiles longitudinally and reported that specimens collected 1–2 weeks prior to an LRTI were significantly enriched with *Moraxella*-dominated profiles compared to all other healthy specimens.

#### Staphylococcus aureus

There was no overall association between *S. aureus* on culture or PCR and LRTI (10 studies) (pooled OR 0.89; 95% CI 0.62–1.26) (Fig. 3; Appendix 3: Fig. S7), or when including only studies with NOS scores ≥7 and > 200 participants (six studies) (Appendix 3: Fig. S8). Subgroup analyses suggested lower prevalence of *S. aureus* in cases versus controls in participants diagnosed with pneumonia versus controls (four studies) (pooled OR 0.69; 95% CI 0.57–0.84) and in younger participants (<24 months) (three studies) (pooled OR 0.76; 95% CI 0.56–1.02) (Appendix 3: Table S4).

There were inconsistent findings for difference between cases and controls at the *Staphylococcus* genus level reported by studies using 16S rRNA gene amplicon sequencing (Fig. 4).

#### Bacteria detected at low prevalence among LRTI cases and controls

*Mycoplasma pneumoniae* (nine studies) and *C. pneumoniae* (seven studies) were detected at low prevalence (Fig. 3) and were similar among cases and controls: *M. pneumoniae* (pooled OR 1.35; 95% CI 0.96–1.90) (Appendix 3: Figs S9, S10; Appendix 3: Table S5); *C. pneumoniae* (pooled OR 0.83; 95% CI 0.59–1.18) (Appendix 3: Figs S11, S12; Appendix 3: Table S6).

Four of the five studies using culture or PCR reported higher prevalence of *Klebsiella* spp. from cases compared to controls (Fig. 3) (pooled OR 2.04; 95% CI 1.17–3.55) (Appendix 3: Figure S13). Limited subgroup analyses could be performed, all of which had ORs >1 (Appendix 3: Table S7).

*Bordetella pertussis, Enterobacter* spp. and *Simkania negevensis* data were only available from ≤3 studies (Fig. 3), and therefore meta-analysis was not done.

#### Bacterial-bacterial co-detection in cases and controls

Ten studies provided bacterial–bacterial co-detection data using culture or PCR from both cases and controls (Appendix 5). Of these, six provided co-detection data at species-level [studies 14,21,32-34 and 36]. Two studies reported statistically significant differences: higher prevalence of *S. pneumoniae*/*H. influenzae* co-detection in cases versus controls [studies 14 and 36]; higher prevalence of *H. influenzae*/*M. catarrhalis* co-detection in cases versus controls [study 36]; higher prevalence of *S. pneumoniae*/*M. catarrhalis* co-detection in controls versus cases [study 36] and higher prevalence of *S. pneumoniae*/*H. influenzae*/*M. catarrhalis* co-detection in controls versus cases [studies 14 and 36] (Appendix 5).

## Discussion

Although *S. pneumoniae* is recognized as the leading bacterial cause of LRTI in children [2,4], several large recent studies have not found evidence of an association between URT carriage and LRTI [12,13]. Our meta-analysis did not identify a significant association, however, after excluding studies reporting more antibiotic treatment prior to sampling in cases than controls, we found a positive association between *S. pneumoniae* URT colonization and LRTI. We further highlight the importance of bacterial density thresholds when investigating the aetiological role of URT bacteria in LRTI, identified by two studies [studies 1 and 36]. These data are supported by all six studies performing 16S rRNA gene amplicon sequencing, which reported higher prevalence of *Streptococcus*-dominated profiles or higher relative abundance of *Streptococcus* genus among cases vs. controls. However, genus level analysis may mask the contribution of streptococcal species other than *Streptococcus pneumoniae* to LRTI. We observed higher prevalence of vaccine type pneumococci from cases compared to controls in eight of 12 studies) (Appendix 4) but could not assess the role of PCV immunisation as five of the eight studies reporting on pneumococci serotypes did not provide details on PCV status of individual participants.

Similar to pneumococcus, the number of Hib associated LRTI deaths among young children has significantly decreased with implementation of Hib-conjugate vaccines [2]. However, the widespread use of Hib-conjugate vaccines has resulted in NTHI strains emerging as a common cause of paediatric LRTI [14]. Our meta-analysis confirmed the association between *H. influenzae* and LRTI, but, since most studies did not serotype strains, we could not assess the role of Hib and NTHI specifically.

While *M. catarrhalis* colonization was variably associated with LRTI, one study reported significant enrichment of *Moraxella*-dominated profiles from specimens collected 1–2 weeks prior to an LRTI [study 43]. Furthermore, co-detection with *H. influenzae* was associated with LRTI [study 36]. Interactions between species may be key to understand the role of *M. catarrhalis* in LRTI. *M. catarrhalis* enhances the development of stable polymicrobial biofilms by promoting the survival of non-typeable *H. influenzae* in the presence of *S. pneumoniae* [15,16]. Interactions have also been demonstrated between other species, for example, an inverse correlation between *S. pneumoniae* and *S. aureus* has been ascribed to hydrogen peroxide production by *S. pneumoniae* [17], inflammation triggered by the pneumococcal pilus [[18], [19], [20]] or cross-reactive antibodies [21]. No overall association between *S. aureus* and LRTI was found.

Other URT bacteria commonly associated with LRTI, particularly in LMICs, include *Mycobacterium tuberculosis*, *B. pertussis*, *E. coli* and *K. pneumoniae* [22]. *M. tuberculosis* may present with acute LRTI and can be detected from nasopharyngeal samples [23]; yet, no studies included in this review screened for *M. tuberculosis* using URT specimens [24,25]. Furthermore, only two studies included *B. pertussis* despite its high burden in LMICs [26].

Five studies screened for *Klebsiella* spp. and meta-analysis showed a higher prevalence among cases. *Klebsiella* spp. have been identified as an important cause of neonatal infections [27] and death [28], particularly in LMICs [29]. PCR was commonly done for *M. pneumoniae* and *C. pneumoniae* [30], yet, their prevalence was low and showed no clear association with LRTI. *M. pneumoniae* and *C. pneumoniae* are more commonly detected from school-aged children, compared with younger children [31]. Sputum, as opposed to NP specimens, has been reported as preferred specimen type to screen for *M. pneumoniae* and *C. pneumoniae* [32].

We identified heterogeneity between studies in the association of the four major bacterial species with LRTI, which may be accounted for by several factors. Firstly, definitions of cases and controls differed between studies. The WHO case definition for pneumonia was used by most studies [33], but lacks specificity [34,35]. Other case definitions included LRTI and bronchiolitis. Cases with bronchiolitis were generally younger than two years of age. Difference in age is likely to be an important source of heterogeneity, given age-related differences in aetiology of LRTI [31]. Controls were primarily classified as asymptomatic. Children with URTI may harbour a different URT microbiota than asymptomatic children [36], hence an ideal population-based control group for LRTI should be balanced between healthy children and those with URTI where dysbiotic URT bacterial communities could control for inflated ORs [37]. Secondly, study populations may contribute to heterogeneity. Hospitalised cases with more severe illness or prior medical treatment may differ from cases diagnosed at community clinics. Hospitalised controls may similarly not reflect the general population. Thirdly, sampling niches with differences in bacterial biomass and composition could also contribute to heterogeneity [38]. The nasopharynx was most frequently sampled [39,40], and shows high overlap with bacterial colonizers of topographically proximate niches such as the anterior nares and the oropharynx [41], yet, the combined study of nasopharyngeal and oropharyngeal niches may be of value [42,43]. Fourthly, age, vaccination coverage, antibiotic treatment, mode of delivery, feeding practices, indoor air pollution, tobacco smoke exposure, crowding, HIV or seasonal changes have been associated with changes in nasopharyngeal microbial communities [[44], [45], [46]]. Only 29 studies (58%) adjusted for confounders, primarily age and season.

A limitation of many of the studies is the targeted detection of only a few bacterial species. A more comprehensive approach towards assessment of the bacterial component is through 16S rRNA gene amplicon sequencing. However, 16S rRNA sequencing may introduce amplification bias and short-read 16S rRNA sequencing used by studies included in this review has limited ability to discriminate at the species level. Metagenomic sequencing may be preferable to allow for de novo identification of pathogens at species-level [47].

This review focused on investigation of URT bacteria, which may not accurately represent the site of infection. LRT specimens, such as induced sputum or bronchoalveolar lavage are likely to provide more direct evidence for aetiology [48,49]. Using transthoracic lung aspirates (TLA) and pleural fluid (PF), the PERCH study identified *S. pneumoniae* and *S. aureus* as predominant pathogens from a select group of children with severe pneumonia [50]. However, sampling of LRT specimens is relatively invasive and requires expertise [51]. Furthermore, LRT specimens are frequently contaminated by URT microbiota [51]. URT specimens are therefore most commonly sampled to investigate LRTI aetiology in children [42,[52], [53], [54]]. It is, however, important to note that although URT specimens are useful to “rule out” causes of LRTI, these specimens are less useful to “rule in” aetiological agents [55].

Studies included in this review identified associations between URT bacteria and LRTI but causality cannot be directly inferred. Furthermore, molecular techniques used by several studies included in this review may improve bacterial detection rates, but do not distinguish between live pathogenic organisms and residual nucleic acid from non-viable organisms, nor do they distinguish between commensal and pathogenic organisms.

This systematic review was limited to bacterial pathogens and did not include studies investigating associations between non-bacterial pathogens and LRTI. The aetiological role of respiratory viruses in LRTI among children under the age of five has recently been reviewed [56]. Furthermore, although viruses have been described as the most common aetiologic agent of LRTI, the severity of disease associated with bacterial infection and increasing antibiotic resistance makes bacterial pneumonia a major public health concern. A previous systematic review examining temporal associations between respiratory viruses and bacteria concluded that viruses both alter bacterial communities in the URT and contribute to bacterial colonization of the LRT [57]. However, the latter review could not quantify the contribution of bacteria to LRTI in the context of viral infection.

Data included in this review were derived from epidemiological studies of association. Such studies have limited ability to address pathogenetic mechanisms. However, they raise important questions around pathogenesis. Does bacterial colonization of the URT always precede LRTI, and how does bacterial density relate to the risk of subsequent LRTI? How does the balance shift between colonization and invasive disease? How does signalling within URT bacterial communities influence progression to LRTI? Further studies aimed at elucidating these pathogenetic process are needed.

## Transparency declaration

The authors declare no conflict of interest. Research reported in this publication was supported by the National Institutes of Health Common Fund, through the 10.13039/100006086Office of Strategic Coordination/10.13039/100000052Office of the NIH Director, 10.13039/100000066National Institute of Environmental Health Sciences and National Human Genome Institute of Health of the 10.13039/100000002National Institutes of Health (H3Africa awards grant numbers U54HG009824 and 1U01AI110466); 10.13039/100000865Bill & Melinda Gates Foundation, Seattle, WA (grant number OPP1017641, OPP1017579). M.P.N. is supported by an Australian National Health and Medical Research Council Investigator Grant (APP1174455). S.C. received funding from the 10.13039/501100001321National Research Foundation South Africa and the L’Oréal-UNESCO For Women in Science South African Young Talents Award. H.J.Z. received funding from the 10.13039/501100001322South African Medical Research Council. The funding bodies had no role in the study design, the collection, analysis or interpretation of the data or the writing of the manuscript.

## Contributors

S.C. was involved in study conception and design, conducted the study searches, screening, data extraction, analysis and interpretation and drafted the final report. K.Y.L.L. and C.M. were involved in screening, data extraction checks and contributed to the final report. H.J.Z. was involved in study conception and design and contributed to the final report. M.P.N. was involved in study conception and design and drafting of the final report. All authors approved the submitted version.
