# Supplementary material for: The association between bacteria colonizing the upper respiratory tract and lower respiratory tract infection in young children: a systematic review and meta-analysis
Source: Clin Microbiol Infect. 2021 Sep;27(9):1262–70. doi: 10.1016/j.cmi.2021.05.034 (PMC8437050; doi:10.1016/j.cmi.2021.05.034)
Supplement: Multimedia component 1 [file mmc1.docx]

**Appendix 1**

**Methodology**

**Protocol registration**

The review protocol is registered with the International Prospective Register of Systematic Reviews (PROSPERO) (CRD42020200544) [1]. We followed the Preferred Reporting Items for Systematic Reviews and Meta-Analyses (PRISMA) criteria for the reporting of systematic reviews [2].

**Search strategy**

We searched MEDLINE (via PubMed), Academic Search Premier, Africa-Wide Information and CINAHL (via EBSCOhost), Scopus and Web of Science for articles reporting on the detection of URT bacteria in children under the age of five years with LRTI and controls. We designed a broad search strategy with guidance from an experienced librarian. We searched MEDLINE via PubMed by selecting “All Fields” and combining medical subject headings (MeSH) and text words related to the research question. We performed the systematic search on 21 July 2020 without publication year and language limitations and exported records to Covidence software [3] for screening and selection of eligible studies.

**Eligibility criteria**

We used the Population, Intervention, Comparison, Outcome and Study design (PICOS) strategy to develop an effective screening strategy (Table 1). We (SC, KYLL and CM) independently performed a two-step selection process: 1) title and abstract screening of deduplicated studies and 2) full text analysis against eligibility criteria outlined in Table 1. Potentially eligible studies for which the full texts could not be assessed for eligibility due to translational issues are provided in Appendix 2. Consensus by a minimum of two reviewers for both screening steps was required. A third reviewer solved discrepancies at each of these steps.

**Appendix 1**

**Methodology (continued)**

**Data extraction**

We extracted data from all eligible studies using a predefined data extraction template. One reviewer (SC) extracted data from all eligible studies. Each of the data extracts were randomly assigned for cross-check by KYLL or CM. Disagreements were resolved by referring to studies and through discussion. We screened supplementary data and contacted corresponding authors if the outcome data in the original study were unclear.

**Risk of bias assessment**

We evaluated studies included in our review based on the following items in the Newcastle-Ottawa Quality Assessment Scale (NOS) for case-control studies: 1) adequate selection of participants (maximum score = 4 points); 2) comparability of cases and controls on the basis of study design or analysis (maximum score = 2 points); and 3) adequate ascertainment of exposures (maximum score = 3 points) [4]. We considered a study to have a low risk of bias if the NOS score obtained was >7.

**Data analysis**

We collated and summarized study characteristics in the form of a narrative synthesis. We used the R package *ggplot* [5][6][7] to present URT bacterial prevalence data (number of participants positive by PCR or culture divided by the number of participants screened) among LRTI cases and controls. We determined the association between the prevalence of URT bacterial species, and the risk of LRTI using pooled odds ratios (ORs) and corresponding confidence intervals (CIs). Pooled ORs were calculated in RevMan [8] using Mantel Haenszel fixed- or random-effects models based on between-study heterogeneity. We used the I^2^ test for inconsistency to measure heterogeneity of data included (heterogeneity was considered relevant if I^2^ > 40%). We excluded studies with potentially overlapping participants from all quantitative analyses to avoid “double counting” aetiological data from a single set of participants. We performed sensitivity analyses for each meta-analysis by excluding studies with high risk of bias (NOS score < 7) and < 200 participants. We considered subgroup analyses in relation to country income groups (low- and lower-middle-income vs. upper-middle- and high-income), laboratory techniques used to screen for URT bacteria (culture vs. PCR), participant age ranges (0-24 months vs. 0-60 months), case definitions used (severe or very severe pneumonia vs. pneumonia vs. LRTI), and antibiotic administration prior to sampling (in cases compared to controls).

**References**

1. Claassen-Weitz S, Lim KY, Mullally C, Zar HJ, Nicol MP. The association between detection of bacteria in the upper respiratory tract with lower respiratory tract infection in young children: protocol for a systematic review [Internet]. PROSPERO. 2020. p. CRD42020200544. Available from: https://www.crd.york.ac.uk/prospero/display_record.php?ID=CRD42020200544

2. Moher D, Liberati A, Tetzlaff J, Altman DG. Preferred reporting items for systematic reviews and meta-analyses: the PRISMA statement. PLoS Med [Internet]. 2009;6(7):e1000097. Available from: https://doi.org/10.1371/journal.pmed.1000097

3. Veritas Health Innovation. Covidence systematic review software [Internet]. Melbourne, Australia; 2014. Available from: www.covidence.org

4. Wells G, Shea B, O’Connell D, Peterson J, Welch V, Losos M, et al. The Newcastle-Ottawa Scale (NOS) for assessing the quality of nonrandomised studies in meta-analyses [Internet]. Available from: http://www.ohri.ca/programs/clinical_epidemiology/oxford.asp

5. Wickham H. ggplot2: Elegant Graphics for Data Analysis [Internet]. Springer-Verlag New York; 2016. Available from: https://ggplot2.tidyverse.org

6. R Core Team. R Foundation for Statistical Computing. R: A language and environment for statistical computing [Internet]. Vienna, Austria; 2018. Available from: http://www.r-project.org/.

7. RStudio. RStudio: Integrated development environment for R [Internet]. Boston, MA; 2012. Available from: http://www.rstudio.org/

8. Review Manager (RevMan) [Computer Program]. Version 5.4. Copenhagen: The Nordic Cochrane Centre, The Cochrane Collaboration, 2020.

**Appendix 1**

**PRISMA checklist**

| **Section/topic** | **#** | **Checklist item** | **Reported** |
| --- | --- | --- | --- |
| **TITLE** | | |  |
| Title | 1 | Identify the report as a systematic review, meta-analysis, or both. | Yes – see title |
| **ABSTRACT** | | |  |
| Structured summary | 2 | Provide a structured summary including, as applicable: background; objectives; data sources; study eligibility criteria, participants, and interventions; study appraisal and synthesis methods; results; limitations; conclusions and implications of key findings; systematic review registration number. | Yes – see abstract |
| **INTRODUCTION** | | |  |
| Rationale | 3 | Describe the rationale for the review in the context of what is already known. | Yes – see introduction |
| Objectives | 4 | Provide an explicit statement of questions being addressed with reference to participants, interventions, comparisons, outcomes, and study design (PICOS). | Yes – see introduction |
| **METHODS** | | |  |
| Protocol and registration | 5 | Indicate if a review protocol exists, if and where it can be accessed (e.g., Web address), and, if available, provide registration information including registration number. | Yes – see methods |
| Eligibility criteria | 6 | Specify study characteristics (e.g., PICOS, length of follow-up) and report characteristics (e.g., years considered, language, publication status) used as criteria for eligibility, giving rationale. | Yes – see methods  Table 1 |
| Information sources | 7 | Describe all information sources (e.g., databases with dates of coverage, contact with study authors to identify additional studies) in the search and date last searched. | Yes – see methods  Appendix 1 |
| Search | 8 | Present full electronic search strategy for at least one database, including any limits used, such that it could be repeated. | Appendix 1 |
| Study selection | 9 | State the process for selecting studies (i.e., screening, eligibility, included in systematic review, and, if applicable, included in the meta-analysis). | Yes – see methods  Appendix 1 |
| Data collection process | 10 | Describe method of data extraction from reports (e.g., piloted forms, independently, in duplicate) and any processes for obtaining and confirming data from investigators. | Yes – see methods  Appendix 1 |

**Appendix 1**

**PRISMA checklist (continued)**

| **Section/topic** | **#** | **Checklist item** | **Reported** |
| --- | --- | --- | --- |
| Data items | 11 | List and define all variables for which data were sought (e.g., PICOS, funding sources) and any assumptions and simplifications made. | Yes – see methods |
| Risk of bias in individual studies | 12 | Describe methods used for assessing risk of bias of individual studies (including specification of whether this was done at the study or outcome level), and how this information is to be used in any data synthesis. | Yes – see methods  Appendix 1 |
| Summary measures | 13 | State the principal summary measures (e.g., risk ratio, difference in means). | Yes – see methods  Appendix 1 |
| Synthesis of results | 14 | Describe the methods of handling data and combining results of studies, if done, including measures of consistency (e.g., I^2^) for each meta-analysis. | Yes – see methods  Appendix 1 |
| Risk of bias across studies | 15 | Specify any assessment of risk of bias that may affect the cumulative evidence (e.g., publication bias, selective reporting within studies). | Yes – see methods |
| Additional analyses | 16 | Describe methods of additional analyses (e.g., sensitivity or subgroup analyses, meta-regression), if done, indicating which were pre-specified. | Yes – see methods  Appendix 1 |
| **RESULTS** | | |  |
| Study selection | 17 | Give numbers of studies screened, assessed for eligibility, and included in the review, with reasons for exclusions at each stage, ideally with a flow diagram. | Yes – see results  Appendix 2 |
| Study characteristics | 18 | For each study, present characteristics for which data were extracted (e.g., study size, PICOS, follow-up period) and provide the citations. | Yes – see results  Appendix 2  Figure 2 |
| Risk of bias within studies | 19 | Present data on risk of bias of each study and, if available, any outcome level assessment (see item 12). | Appendix 2 |
| Results of individual studies | 20 | For all outcomes considered (benefits or harms), present, for each study: (a) simple summary data for each intervention group (b) effect estimates and confidence intervals, ideally with a forest plot. | Yes – see results  Appendix 3  Figures 3-4 |

**Appendix 1**

**PRISMA checklist (continued)**

| **Section/topic** | **#** | **Checklist item** | **Reported** |
| --- | --- | --- | --- |
| Synthesis of results | 21 | Present results of each meta-analysis done, including confidence intervals and measures of consistency. | Yes – see results  Appendix 3  Figures 3-4 |
| Risk of bias across studies | 22 | Present results of any assessment of risk of bias across studies (see Item 15). | Data not shown |
| Additional analysis | 23 | Give results of additional analyses, if done (e.g., sensitivity or subgroup analyses, meta-regression [see Item 16]). | Yes – see results  Appendix 3 |
| **DISCUSSION** | | |  |
| Summary of evidence | 24 | Summarize the main findings including the strength of evidence for each main outcome; consider their relevance to key groups (e.g., healthcare providers, users, and policy makers). | Yes – see discussion |
| Limitations | 25 | Discuss limitations at study and outcome level (e.g., risk of bias), and at review-level (e.g., incomplete retrieval of identified research, reporting bias). | Yes – see discussion |
| Conclusions | 26 | Provide a general interpretation of the results in the context of other evidence, and implications for future research. | Yes – see discussion |
| **FUNDING** | | |  |
| Funding | 27 | Describe sources of funding for the systematic review and other support (e.g., supply of data); role of funders for the systematic review. | Yes – see funding section |

From: Moher D, Liberati A, Tetzlaff J, Altman DG, The PRISMA Group (2009). Preferred Reporting Items for Systematic Reviews and Meta-Analyses: The PRISMA Statement. PLoS Med 6(7): e1000097. doi:10.1371/journal.pmed1000097 (www.prisma-statement.org)

**Appendix 1**

**Search Strategy**

**MEDLINE (via PubMed):**

**1923 to July 2020**

(((((((((((("Bronchiolitis"[Mesh]) OR "Pneumonia"[Mesh]) OR Bronchiolitis) OR pneumonia) OR lower respiratory tract infection) OR lung infection) OR lung inflammation) OR pertussis) OR pulmonary inflammation))

AND

(((((((((((((("Bacteria"[Mesh]) OR "Haemophilus influenzae"[Mesh]) OR "Microbiota"[Mesh]) OR "Moraxella catarrhalis"[Mesh]) OR "Staphylococcus aureus"[Mesh]) OR "Streptococcus pneumoniae"[Mesh]) OR Bacteria) OR Haemophilus influenzae) OR microbiota) OR microflora) OR microbiome) OR Moraxella catarrhalis) OR Staphylococcus aureus) OR Streptococcus pneumoniae))

AND

(((((((("Nasopharynx"[Mesh]) OR "Oropharynx"[Mesh]) OR Nares) OR nasal) OR nasopharynx) OR nasopharyngeal) OR oropharynx) OR oropharyngeal))

AND

(((((((((((((((("Infant"[Mesh]) OR "Child"[Mesh]) OR "Pediatrics"[Mesh]) OR child) OR children) OR early life) OR paediatrics) OR paediatric) OR pediatrics) OR pediatric) OR preschools) OR preschool) OR pre-schools) OR pre-school) OR infants) OR infant)

**Appendix 1**

**Search Strategy (continued)**

**Academic Search Premier, Africa-Wide Information and CINAHL (via EBSCOhost):**

**1969 to July 2020**

Bronchiolitis OR lower respiratory tract infection* OR lung infection OR lung inflammation OR pertussis OR pneumonia OR pulmonary inflammation

AND

Bacteria OR Haemophilus influenzae OR microbiota OR microflora OR microbiome OR Moraxella catarrhalis OR Staphylococcus aureus OR Streptococcus pneumoniae

AND

Nares OR nasal OR nasopharynx OR nasopharyngeal OR oropharynx OR oropharyngeal

AND

child* OR early life OR infant* OR pediatric* OR paediatric* OR preschool* OR pre-school*

**Appendix 1**

**Search Strategy (continued)**

**Scopus:**

**1960 to July 2020**

( ( TITLE-ABS-KEY ( bronchiolitis ) OR TITLE-ABS-KEY ( lower AND respiratory AND tract AND infection* ) OR TITLE-ABS-KEY ( lung AND infection ) OR TITLE-ABS-KEY ( lung AND inflammation ) OR TITLE-ABS-KEY ( pertussis ) OR TITLE-ABS-KEY ( pneumonia ) OR TITLE-ABS-KEY ( pulmonary AND inflammation ) ) )

AND

( ( TITLE-ABS-KEY ( bacteria ) OR TITLE-ABS-KEY ( haemophilus AND influenzae ) OR TITLE-ABS-KEY ( microbiota ) OR TITLE-ABS-KEY ( microflora ) OR TITLE-ABS-KEY ( microbiome ) OR TITLE-ABS-KEY ( moraxella AND catarrhalis ) OR TITLE-ABS-KEY ( staphylococcus AND aureus ) OR TITLE-ABS-KEY ( streptococcus AND pneumoniae ) ) )

AND

( ( TITLE-ABS-KEY ( nares ) OR TITLE-ABS-KEY ( nasal ) OR TITLE-ABS-KEY ( nasopharynx ) OR TITLE-ABS-KEY ( nasopharyngeal ) OR TITLE-ABS-KEY ( oropharynx ) OR TITLE-ABS-KEY ( oropharyngeal ) ) )

AND

( ( TITLE-ABS-KEY ( child ) OR TITLE-ABS-KEY ( early AND life ) OR TITLE-ABS-KEY ( infant* ) OR TITLE-ABS-KEY ( pediatric* ) OR TITLE-ABS-KEY ( paediatric* ) OR TITLE-ABS-KEY ( preschool* ) OR TITLE-ABS-KEY ( pre-school* ) ) )

**Appendix 1**

**Search Strategy (continued)**

**Web of Science:**

**1962 to July 2020**

TOPIC: (Bronchiolitis) OR TOPIC: (lower respiratory tract infection*) OR TOPIC: (lung infection) OR TOPIC: (lung inflammation) OR TOPIC: (pertussis) OR TOPIC: (pneumonia) OR TOPIC: (pulmonary inflammation)

AND

TOPIC: (Bacteria) OR TOPIC: (Haemophilus influenzae) OR TOPIC: (microbiota) OR TOPIC: (microflora) OR TOPIC: (microbiome) OR TOPIC: (Moraxella catarrhalis) OR TOPIC: (Staphylococcus aureus) OR TOPIC: (Streptococcus pneumoniae)

AND

TOPIC: (nares) OR TOPIC: (nasal) OR TOPIC: (nasopharynx) OR TOPIC: (nasopharyngeal) OR TOPIC: (oropharynx) OR TOPIC: (oropharyngeal)

AND

TOPIC: (child*) OR TOPIC: (early life) OR TOPIC: (infant*) OR TOPIC: (pediatric*) OR TOPIC: (paediatric*) OR TOPIC: (preschool*) OR TOPIC: (pre-school*)

**Appendix 1**

**Definitions**

| Term | Definition |
| --- | --- |
| Respiratory tract infection (RTI) | Infection of the respiratory tract without information on the site of infection (upper or lower) |
| Lower respiratory tract infection (LRTI) | Acute infection of the lower respiratory tract including pneumonia, bronchiolitis and pertussis |
| Upper respiratory tract infection (URTI) | Infection of the upper respiratory tract without reports of infection of the lower respiratory tract |
| Lower respiratory tract (LRT) specimen | A specimen from the lower respiratory tract including larynx, trachea, or lungs |
| Upper respiratory tract (URT) specimen | A specimen from the upper respiratory tract including nasopharynx, oropharynx, or nasal passages |
| Single-centre publication | Publication including participants from a single city/town/village/area/site within a single country. |
| Multi-centre publication | Publication including participants from multiple cities/towns/villages/areas/sites within a single country or across multiple countries. |
| Microbiota profiling groups | Specimens classified based on short fragment 16S rRNA gene amplicon profiles dominated by one or more than one genus/operational taxonomic unit (OTU)/amplicon sequence variant (ASV) |
| Same Child Control cohort | LRTI specimen matched to non-LRTI specimen from the same child |
| Different Child Control cohort | LRTI specimen matched to non-LRTI specimen from a different child |

**Appendix 1**

**Data extraction template**

| Domain | Variable | | Specification |
| --- | --- | --- | --- |
| Study characteristics | Record number |  | ….. |
|  | Study |  | First author, publication year (Study name) |
|  | Country |  | ….. |
|  | City / Town / Village |  | ….. |
|  | Geographic coordinates |  | ….. |
|  | Design |  | CSCC / LCC |
|  | Age range enrolled (M) |  | ….. |
|  | Specimen(s) |  | ANS / NPA / NPS / NS / NW /OPS |
|  | Diagnostic test(s) | Primary test(s) | ….. |
|  |  | Additional test(s) | ….. |
|  | Microbe(s) screened (N) | Bacteria | >1 |
|  |  | Viruses | >0 |
|  |  | Fungi | >0 |
|  | Population size (N) |  | ….. |
|  | Case group | Number of participants (%) | ….. |
|  |  | Definition (criteria) | ALRI (…..) / ARI (…..) / B (…..) / LRTI (…..) / P (…..) / RSV-B (..…)/ RSV-infection (…..) / SARI (…..) / SP (…..) / VSP (…..) |
|  |  | Proportion male participants | ….. |
|  |  | Age (M) | ….. |
|  |  | Setting | CO / ED / H / O / PHC |
|  |  | Proportion of participants with antibiotic administration prior to sampling | ….. |
|  |  | Vaccination history (%) | ….. |

**Appendix 1**

**Data extraction template (continued)**

| Domain | | Variable | | Specification | |
| --- | --- | --- | --- | --- | --- |
| Study characteristics | Control group | | Number of participants (%) | | ….. |
|  |  |  | Definition (criteria) | | Community controls (…..) / Healthy controls (…..) / No LRTI / No P (…..) / No RI (…..) / URTI (…..) |
|  |  |  | Proportion male participants | | …… |
|  |  |  | Age (M) | | ….. |
|  |  |  | Setting | | CO / ED / H / O / PHC |
|  |  |  | Proportion of participants with antibiotic administration prior to sampling | | ….. |
|  |  |  | Vaccination history (%) | | ….. |
|  | Adjustment for confounders | |  | | ….. |
| URT bacterial prevalence data (bacterial species +/- viruses +/- fungi) from both LRTI cases and controls generated using culture and PCR | Record number | |  | | ….. |
|  | Study | |  | | First author, publication year (Study name) |
|  | Bacterial spp. | | Cases (n) | | ….. |
|  |  |  | Cases (N) | | ….. |
|  |  |  | Cases (%) | | ….. |
|  |  |  | Controls (n) | | ….. |
|  |  |  | Controls (N) | | ….. |
|  |  |  | Controls (%) | | ….. |
|  |  |  | p-value | | ….. |
|  |  |  | Adjusted odds ratio | | ….. |
|  | Viral spp. / Fungal spp. | | Cases (n) | | ….. |
|  |  |  | Cases (N) | | ….. |
|  |  |  | Cases (%) | | ….. |
|  |  |  | Controls (n) | | ….. |
|  |  |  | Controls (N) | | ….. |

**Appendix 1**

**Data extraction template (continued)**

| Domain | | Variable | | Specification |
| --- | --- | --- | --- | --- |
| URT bacterial prevalence data (bacterial species +/- viruses +/- fungi) from both LRTI cases and controls generated using culture and PCR | |  | Controls (%) | ….. |
|  |  |  | p-value | ….. |
|  |  |  | Adjusted odds ratio | ….. |
|  |  | Co-detection (bacterial-bacterial / bacterial-viral / bacterial-fungal) | Cases (n) | ….. |
|  |  |  | Cases (N) | ….. |
|  |  |  | Cases (%) | ….. |
|  |  |  | Controls (n) | ….. |
|  |  |  | Controls (N) | ….. |
|  |  |  | Controls (%) | ….. |
|  |  |  | p-value | ….. |
|  |  |  | Adjusted odds ratio | ….. |
| URT bacterial prevalence data (genus-dominated profiles) from both LRTI cases and controls generated using 16S rRNA gene sequencing | Record number | |  | ….. |
|  | Study | |  | First author, publication year (Study name) |
|  | Microbiota profiling group / genus | | Cases (n) | ….. |
|  |  |  | Cases (N) | ….. |
|  |  |  | Cases (%) | ….. |
|  |  |  | Controls (n) | ….. |
|  |  |  | Controls (N) | ….. |
|  |  |  | Controls (%) | ….. |
|  |  |  | p-value | ….. |
|  |  |  | Adjusted odds ratio | ….. |
|  | Reports on microbiota diversity / richness | |  | ….. |
| URT bacterial prevalence data (bacterial serotypes) from both LRTI cases and controls generated using serotype analysis | Record number | |  | ….. |
|  | Study | |  | First author, publication year (Study name) |
|  | Serotypes screened | |  | ….. |
|  | Results | |  | ….. |
|  | Vaccination reports | |  | ….. |

CSCC - Cross-Sectional Case-Control; LCC - Longitudinal Case-Control

ANS - Anterior nasal swab; NPA - Nasopharyngeal aspirates; NPS - Nasopharyngeal swabs; NS - Nasal swabs; NW - Nasal wash; OPS - Oropharyngeal swab

ALRI - Acute Lower Respiratory Infection; ARI - Acute Respiratory Infection; B - Bronchiolitis; LRTI - Lower Respiratory Tract Infection; P - Pneumonia; RI - Respiratory Infection; RSV-B - Respiratory Syncytial Virus Bronchiolitis; SARI - Severe Acute Respiratory Infection; SP - Severe Pneumonia; VSP - Very Severe Pneumonia

CO - Community; ED - Emergency Department; H - Hospital; O - Outpatient; PHC - Primary Healthcare Clinics (including immunisation and study clinics)
