# Supplementary material for: The association between bacteria colonizing the upper respiratory tract and lower respiratory tract infection in young children: a systematic review and meta-analysis
Source: Clin Microbiol Infect. 2021 Sep;27(9):1262–70. doi: 10.1016/j.cmi.2021.05.034 (PMC8437050; doi:10.1016/j.cmi.2021.05.034)
Supplement: Multimedia component 2 [file mmc2.docx]

**Appendix 2**

**Flow diagram of the selection process of studies included in the review**

CAS - Childhood Asthma Study; GABRIEL - Global Approach to Biological Research, Infectious diseases and Epidemics in Low-income countries; PERCH - Pneumonia Etiology Research for Child Health

**Appendix 2**

**Potentially eligible studies for which the full texts could not be assessed for eligibility due to translational issues**

1. Savitskaia KI, Vorob’ev AA, Sinitsina GG. Microflora of the upper respiratory tract in young children under normal conditions and in respiratory tract diseases. Microbial count of the upper respiratory tract in healthy children and in children with pneumonia. Zh Mikrobiol Epidemiol Immunobiol. 1986;Dec(12):24–31.

2. Savitskaia KI, Vorob’ev AA, Solodilova OE. Microflora of the upper respiratory tract of normal young children and in respiratory tract diseases. Normal microflora and dysbiosis at different levels of the upper respiratory tract in healthy children and pneumonia patients. Zh Mikrobiol Epidemiol Immunobiol. 1987;Jan(1):22–9.

3. Passauer I, Beckmann C, Wiesner P. Microbiological studies in children in nurseries (Study A). Padiatr Grenzgeb. 1991;30(4):295–303.

4. Sequeira MD, Zerbini E, Imaz MS, Veronessi I, Latini O, Cociglio R, et al. Etiology of acute lower respiratory tract infections among children younger than 5 years old in Santa Fe. Med (B Aires). 1997;57(2):191–9.

5. Srugo I, Chystiakov I, Cohen E, Tal Y, Jaffe M. Nasopharyngeal colonization with Streptococcus pneumoniae in pediatric respiratory infections. Harefuah. 1998;135(9):350–4.

6. Wang F, Zhu D, Wu W. Bacterial resistance in streptococcus pneumoniae. Zhonghua Yi Xue Za Zhi. 2000;80(10):749–52.

7. Kakinohana S, Hamabata H, Higa N, Nakasone N. Pathogenic bacteria in the nasal vestivulum of children with acute respiratory tract infection. Kansenshogaku zasshi. 2001;75(2):124–32.

8. Aran A, Fraser D, Dagan R. Characteristics of nasopharyngeal carriage of Streptococcus pneumoniae in children during acute respiratory disease. Harefuah. 2001;140(4):300–5.

9. Chen ZM, Wang TL, Shang SQ, Li JP. Study on application of PCR in the diagnosis of Haemophilus influenzae pneumonia. Zhejiang Da Xue Xue Bao Yi Xue Ban. 2002;31(1):47–50.

10. Uno Y. Investigate of nasopharyngeal flora in infants and children with influenza. Kansenshogaku Zasshi. 2003;77(12):1024–31.

11. Wang H, Yu YS, Liu Y, Li HY, Hu BJ, Sun ZY, et al. Resistance surveillance of common community respiratory pathogens isolated in China, 2002 - 2003. Zhonghua Jie He He Hu Xi Za Zhi. 2004;27(3):155–60.

12. Metel’skaia VA, Aleshkin BA, Voropaeva EA, Karaulov A V, Nesvizhskiĭ I V, Afanas’ev SS, et al. Colonization resistance and immunological reactivity of children’s oropharyngeal mucosa in health and bronchopulmonary pathology. Vestn Ross Akad Med Nauk. 2010;(7):10–5.

13. Kholodok GN, Morozova N V, Alekseeva IN, Stukun EA, Krakovskaia A V, Morozova OI, et al. Rate of identification and characteristic of Streptococcus pneumoniae isolated from carriers and ill children in Khabarovsk region. Zh Mikrobiol Epidemiol Immunobiol. 2010;(4):92–6.

14. Kholodok GN, Alekseeva IN, Strel’nikova N V, Kozlov VK. Colonization properties of opportunistic bacteria isolated from children with pneumonia. Zh Mikrobiol Epidemiol Immunobiol. 2017;(2):17–25.

**Appendix 2**

**Potentially eligible studies for which the full texts could not be assessed for eligibility due to translational issues (continued)**

15. Yatsishina SB, Spichak T V, Kim SS, Uchaykin VF, Pokrovskiy VI. Revealing of respiratory viruses and atypical bacteria in children with pneumonia and healthy children for ten years of observation. Pediatriya. 2016;95(2):43–50.

**Appendix 2**

**Reference list of eligible studies included in the review**

1. The Pneumonia Etiology Research for Child Health (PERCH) Study Group. Causes of severe pneumonia requiring hospital admission in children without HIV infection from Africa and Asia: the PERCH multi-country case-control study. Lancet [Internet]. 2019;394(10200):757–79. Available from: https://www.thelancet.com/journals/lancet/article/PIIS0140-6736(19)30721-4/fulltext

2. Park DE, Baggett HC, Howie SRC, Shi Q, Watson NL, Brooks WA, et al. Colonization density of the upper respiratory tract as a predictor of pneumonia - Haemophilus influenzae, Moraxella catarrhalis, Staphylococcus aureus, and Pneumocystis jirovecii. Clin Infect Dis [Internet]. 2017;64(Suppl 3):S328–36. Available from: https://doi.org/10.1093/cid/cix104

3. Barger-Kamate B, Knoll MD, Kagucia EW, Prosperi C, Baggett HC, Brooks WA, et al. Pertussis-associated pneumonia in infants and children from low-and middle-income countries participating in the perch study. Clin Infect Dis [Internet]. 2016;63(Suppl 4):S187–96. Available from: https://doi.org/10.1093/cid/ciw546

4. Baggett HC, Watson NL, Knoll MD, Brooks WA, Feikin DR, Hammitt LL, et al. Density of upper respiratory colonization with Streptococcus pneumoniae and its role in the diagnosis of pneumococcal pneumonia among children aged <5 years in the PERCH study. Clin Infect Dis [Internet]. 2017;64(Suppl 3):S317–27. Available from: https://doi.org/10.1093/cid/cix100

5. Piralam B, Prosperi C, Thamthitiwat S, Bunthi C, Sawatwong P, Sangwichian O, et al. Pneumococcal colonization prevalence and density among Thai children with severe pneumonia and community controls. PLoS One [Internet]. 2020;15(4):e0232151. Available from: http://dx.doi.org/10.1371/journal.pone.0232151

6. Camelo IY, Mwananyanda LM, Thea DM, Seidenberg P, Gill CJ, Weinstein JR. A Tale of 2 Pneumos: The Impact of Human Immunodeficiency Virus Exposure or Infection Status on Pediatric Nasopharyngeal Carriage of Streptococcus pneumoniae and Pneumocystis jiroveci: A Nested Case Control Analysis From the Pneumonia Etiology Research In. Clin Infect Dis [Internet]. 2020 Feb 15;1–9. Available from: https://doi.org/10.1093/cid/ciaa164

7. Jroundi I, Mahraoui C, Benmessaoud R, Moraleda C, Munoz Almagro C, Seffar M, et al. Streptococcus pneumoniae carriage among healthy and sick pediatric patients before the generalized implementation of the 13-valent pneumococcal vaccine in Morocco from 2010 to 2011. J Infect Public Health [Internet]. 2016;10(2):165–70. Available from: http://dx.doi.org/10.1016/j.jiph.2016.02.012

8. Praygod G, Mukerebe C, Magawa R, Jeremiah K, Török ME. Indoor Air Pollution and Delayed Measles Vaccination Increase the Risk of Severe Pneumonia in Children: Results from a Case-Control Study in Mwanza, Tanzania. PLoS One [Internet]. 2016;11(8):e0160804. Available from: https://doi.org/10.1371/journal.pone.0160804

9. Kumar S, Awasthi S, Jain A, Srivastava RC. Blood zinc levels in children hospitalized with severe pheumonia: A case control study. Indian Pediatr [Internet]. 2004;41(5):486–91. Available from: http://www.indianpediatrics.net/may2004/may-486-491.htm

10. Hammitt LL, Kazungu S, Morpeth SC, Gibson DG, Mvera B, Brent AJ, et al. A preliminary study of pneumonia etiology among hospitalized children in Kenya. Clin Infect Dis [Internet]. 2012;54(Suppl 2). Available from: https://doi.org/10.1093/cid/cir1071

**Appendix 2**

**Reference list of eligible studies included in the review (continued)**

11. Feikin DR, Njenga MK, Bigogo G, Aura B, Aol G, Audi A, et al. Viral and bacterial causes of severe acute respiratory illness among children aged less than 5 years in a high malaria prevalence area of Western Kenya, 2007-2010. Pediatr Infect Dis J [Internet]. 2013;32(1):2007–10. Available from: https://journals.lww.com/pidj/Abstract/2013/01000/Viral_and_Bacterial_Causes_of_Severe_Acute.10.aspx

12. Greenberg D, Givon-lavi N, Newman N, Bar-ziv J, Dagan R. Nasopharyngeal Carriage of Individual Streptococcus pneumoniae Serotypes During Pediatric Pneumonia as a Means to Estimate Serotype Disease Potential. Pediatr Infect Dis J [Internet]. 2011;30(3):227–33. Available from: https://journals.lww.com/pidj/Abstract/2011/03000/Nasopharyngeal_Carriage_of_Individual.11.aspx

13. Adebanjo T, Lessa FC, Mucavele H, Moiane B, Chauque A, Pimenta F, et al. Pneumococcal carriage and serotype distribution among children with and without pneumonia in Mozambique, 2014-2016. PLoS One [Internet]. 2018;13(6):2014–6. Available from: https://doi.org/10.1371/journal.pone.0199363

14. Ngocho JS, Minja L, van der Gaast - de Jongh CE, Rahamat-Langendoen JC, Langereis JD, Mmbaga BT, et al. Viral-bacterial (co-) occurrence in the upper airways and the risk of childhood pneumonia in resource-limited settings. J Infect [Internet]. 2020;81:213–20. Available from: https://doi.org/10.1016/j.jinf.2020.06.013

15. Rey LC, Wolf B, Moreira JLB, Milatovic D, Verhoef J, Farhat CK. Antimicrobial susceptibility and serotypes of nasopharyngeal Streptococcus pneumoniae in children with pneumonia and in children attending day-care centres in Fortaleza, Brazil. Int J Antimicrob Agents [Internet]. 2002;20(2):86–92. Available from: https://doi.org/10.1016/S0924-8579(02)00128-0

16. Wolf B, Rey LC, Moreira LB, Milatovic D, Fleer A, Verhoef J, et al. Carriage of gram-negative bacilli in young Brazilian children with community-acquired pneumonia. Int J Infect Dis [Internet]. 2001;5(3):155–9. Available from: https://www.ijidonline.com/article/S1201-9712(01)90091-8/pdf

17. Weber MW, Gopalakrishna G, Awomoyi A, Cunningham A, Adegbola RA, Falade AG, et al. The role of Chlamydia pneumoniae in acute respiratory tract infections in young children in The Gambia, West Africa. Ann Trop Paediatr [Internet]. 2006;26(2):87–94. Available from: https://doi.org/10.1179/146532806X107412

18. Bénet T, Sylla M, Messaoudi M, Picot VS, Telles JN, Diakite AA, et al. Etiology and factors associated with pneumonia in children under 5 years of age in Mali: A prospective case-control study. PLoS One [Internet]. 2015;10(12):1–15. Available from: https://doi.org/10.1371/journal.pone.0145447

19. Bénet T, Sánchez Picot V, Messaoudi M, Chou M, Eap T, Wang J, et al. Microorganisms Associated with Pneumonia in Children <5 Years of Age in Developing and Emerging Countries: The GABRIEL Pneumonia Multicenter, Prospective, Case-Control Study. Clin Infect Dis [Internet]. 2017;65(4):604–12. Available from: https://doi.org/10.1093/cid/cix378

20. Dananché C, Paranhos-Baccalà G, Messaoudi M, Sylla M, Awasthi S, Bavdekar A, et al. Serotypes of Streptococcus pneumoniae in Children Aged <5 Years Hospitalized With or Without Pneumonia in Developing and Emerging Countries: A Descriptive, Multicenter Study. Clin Infect Dis [Internet]. 2020;70(5):875–83. Available from: https://doi.org/10.1093/cid/ciz277

**Appendix 2**

**Reference list of eligible studies included in the review (continued)**

21. Zar HJ, Barnett W, Stadler A, Gardner-Lubbe S, Myer L, Nicol MP. Aetiology of childhood pneumonia in a well vaccinated South African birth cohort: A nested case-control study of the Drakenstein Child Health Study. Lancet Respir Med [Internet]. 2016;4(6):463–72. Available from: http://dx.doi.org/10.1016/S2213-2600(16)00096-5

22. Kelly MS, Surette MG, Smieja M, Pernica JM, Rossi L, Luinstra K, et al. The Nasopharyngeal Microbiota of Children with Respiratory Infections in Botswana. Pediatr Infect Dis J [Internet]. 2017;36(9):e211–8. Available from: https://journals.lww.com/pidj/Abstract/2017/09000/The_Nasopharyngeal_Microbiota_of_Children_With.6.aspx

23. Levine OS, Liu G, Garman RL, Dowell SF, Yu S, Yang YH. Haemophilus influenzae type B and Streptococcus pneumoniae as causes of pneumonia among children in Beijing, China. Emerg Infect Dis [Internet]. 2000;6(2):165–70. Available from: https://wwwnc.cdc.gov/eid/article/6/2/00-0209_article

24. Sutcliffe CG, Shet A, Varghese R, Veeraraghavan B, Manoharan A, Wahl B, et al. Nasopharyngeal carriage of Streptococcus pneumoniae serotypes among children in India prior to the introduction of pneumococcal conjugate vaccines: A cross-sectional study. BMC Infect Dis [Internet]. 2019;19(1):1–12. Available from: https://bmcinfectdis.biomedcentral.com/articles/10.1186/s12879-019-4254-2

25. Mogdasy MC, Camou T, Fajardo C, Hortal M. Colonizing and invasive strains of Streptococcus pneumoniae in Uruguayan children: Type distribution and patterns of antibiotic resistance. Pediatr Infect Dis J [Internet]. 1992;11(8):648–52. Available from: https://www.researchgate.net/publication/21632800_Colonizing_and_invasive_strains_of_Streptococcus_pneumoniae_in_Uruguayan_children_Type_distribution_and_patterns_of_antibiotic_resistance

26. Macasaet FF, Kidd PA, Bolano CR, Wenner HA. The etiology of acute respiratory infections III. The role of viruses and bacteria. J Pediatr [Internet]. 1968;72(6):829–39. Available from: https://doi.org/10.1016/S0022-3476(68)80436-6

27. Bhuiyan MU, Snelling TL, West R, Lang J, Rahman T, Granland C, et al. The contribution of viruses and bacteria to community-acquired pneumonia in vaccinated children: a case-control study. Thorax [Internet]. 2019;74:261–9. Available from: http://dx.doi.org/10.1136/thoraxjnl-2018-212096

28. Liu G, Talkington DF, Fields BS, Levine OS, Yang Y, Tondella MLC. Chlamydia pneumoniae and Mycoplasma pneumoniae in young children from China with community-acquired pneumonia. Diagn Microbiol Infect Dis [Internet]. 2005;52(1):7–14. Available from: https://doi.org/10.1016/j.diagmicrobio.2005.01.005

29. Palmu AA, Ware RS, Lambert SB, Sarna M, Bialasiewicz S, Seib KL, et al. Nasal swab bacteriology by PCR during the first 24-months of life: A prospective birth cohort study. Pediatr Pulmonol [Internet]. 2019;54(3):289–96. Available from: https://doi.org/10.1002/ppul.24231

30. Anh DD, Huong PLT, Watanabe K, Nguyet NT, Ahn NTH, Thi NT, et al. Increased rates of intense nasopharyngeal bacterial colonization of Vietnamese children with radiological pneumonia. Tohoku J Exp Med [Internet]. 2007;213(2):167–72. Available from: https://doi.org/10.1620/tjem.213.167

**Appendix 2**

**Reference list of eligible studies included in the review (continued)**

31. Bhuyan GS, Hossain MA, Sarker SK, Rahat A, Islam MT, Haque TN, et al. Bacterial and viral pathogen spectra of acute respiratory infections in under-5 children in hospital settings in Dhaka city. PLoS One [Internet]. 2017;12(3):1–21. Available from: https://doi.org/10.1371/journal.pone.0174488

32. Mastro TD, Nomani NK, Ishaq Z, Ghafoor A, Shaukat NF, Esko E, et al. Use of nasopharyngeal isolates of Streptococcus pneumoniae and Haemophilus influenzae from children in Pakistan for surveillance for antimicrobial resistance. Pediatr Infect Dis J. 1993;12(10):824–30.

33. Vathanophas K, Sangchai R, Raktham S, Pariyanonda A, Thangsuvan J, Bunyaratabhandu P, et al. A Community-Based Study of Acute Respiratory Tract Infection in Thai Children. Rev Infect Dis [Internet]. 1990;12(8):S957–65. Available from: https://doi.org/10.1093/clinids/12.Supplement_8.S957

34. Smith-Vaughan HC, Binks MJ, Beissbarth J, Chang AB, McCallum GB, Mackay IM, et al. Bacteria and viruses in the nasopharynx immediately prior to onset of acute lower respiratory infections in Indigenous Australian children. Eur J Clin Microbiol Infect Dis [Internet]. 2018;37(9):1785–94. Available from: https://link.springer.com/article/10.1007/s10096-018-3314-7

35. Coles C., Sherchand J., Khatry S., Katz J, LeClerq S., Mullany L., et al. Nasopharyngeal carriage of S. pneumoniae among young children in rural Nepal. Trop Med Int Heal [Internet]. 2009;14(9):1025–33. Available from: https://onlinelibrary.wiley.com/doi/full/10.1111/j.1365-3156.2009.02331.x

36. Vu HTT, Yoshida LM, Suzuki M, Nguyen HAT, Nguyen CDL, Nguyen ATT, et al. Association between nasopharyngeal load of Streptococcus pneumoniae, viral coinfection, and radiologically confirmed pneumonia in Vietnamese children. Pediatr Infect Dis J [Internet]. 2011;30(1):11–8. Available from: https://journals.lww.com/pidj/Abstract/2011/01000/Association_Between_Nasopharyngeal_Load_of.6.aspx

37. Bezerra PGM, Britto MCA, Correia JB, Duarte M do CMB, Fonceca AM, Rose K, et al. Viral and Atypical Bacterial Detection in Acute Respiratory Infection in Children Under Five Years. PLoS One [Internet]. 2011;6(4):e18928. Available from: https://journals.plos.org/plosone/article?id=10.1371/journal.pone.0018928

38. Suárez-Arrabal MC, Mella C, Lopez SM, Brown N V., Hall MW, Hammond S, et al. Nasopharyngeal bacterial burden and antibiotics: Influence on inflammatory markers and disease severity in infants with respiratory syncytial virus bronchiolitis. J Infect [Internet]. 2015;71(4):458–69. Available from: https://www.journalofinfection.com/article/S0163-4453(15)00221-2/fulltext

39. Kumar S, Kohlhoff SA, Gelling M, Roblin PM, Kutlin A, Kahane S, et al. Infection with Simkania negevensis in Brooklyn, New York. Pediatr Infect Dis J. 2005;24(11):989–92.

40. Kahane S, Greenberg D, Friedman MG, Haikin H, Dagan R. High Prevalence of “Simkania Z,” a Novel Chlamydia‐like Bacterium, in Infants with Acute Bronchiolitis. J Infect Dis [Internet]. 1998;177(5):1425–9. Available from: https://doi.org/10.1086/517830

41. Man WH, van Houten MA, Mérelle ME, Vlieger AM, Chu MLJN, Jansen NJG, et al. Bacterial and viral respiratory tract microbiota and host characteristics in children with lower respiratory tract infections: a matched case-control study. Lancet Respir Med [Internet]. 2019;7(5):417–26. Available from: https://doi.org/10.1016/S2213-2600(18)30449-1

**Appendix 2**

**Reference list of eligible studies included in the review (continued)**

42. Teo SM, Mok D, Pham K, Kusel M, Serralha M, Troy N, et al. The Infant Nasopharyngeal Microbiome Impacts Severity of Lower Respiratory Infection and Risk of Asthma Development. Cell Host Microbe [Internet]. 2015;17:1–12. Available from: http://linkinghub.elsevier.com/retrieve/pii/S1931312815001250

43. Teo SM, Tang HHF, Mok D, Judd LM, Watts SC, Pham K, et al. Airway Microbiota Dynamics Uncover a Critical Window for Interplay of Pathogenic Bacteria and Allergy in Childhood Respiratory Disease. Cell Host Microbe [Internet]. 2018;24(3):341-352.e5. Available from: https://doi.org/10.1016/j.chom.2018.08.005

44. De Steenhuijsen Piters WAA, Heinonen S, Hasrat R, Bunsow E, Smith B, Suarez-Arrabal MC, et al. Nasopharyngeal microbiota, host transcriptome, and disease severity in children with respiratory syncytial virus infection. Am J Respir Crit Care Med [Internet]. 2016;194(9):1104–15. Available from: https://doi.org/10.1164/rccm.201602-0220OC

45. Hasegawa K, Linnemann RW, Mansbach JM, Ajami NJ, Espinola J a., Petrosino JF, et al. Nasal Airway Microbiota Profile and Severe Bronchiolitis in Infants: A Case-control Study. Pediatr Infect Dis J [Internet]. 2017;36(11):1044–51. Available from: https://www.ncbi.nlm.nih.gov/pmc/articles/PMC5479744/

46. Ederveen THA, Ferwerda G, Ahout IM, Vissers M, de Groot R, Boekhorst J, et al. Haemophilus is overrepresented in the nasopharynx of infants hospitalized with RSV infection and associated with increased viral load and enhanced mucosal CXCL8 responses. Microbiome [Internet]. 2018;6(10):1–13. Available from: https://link.springer.com/article/10.1186/s40168-017-0395-y

47. Hu Q, Dai W, Zhou Q, Fu D, Zheng Y, Wang W, et al. Dynamic oropharyngeal and faecal microbiota during treatment in infants hospitalized for bronchiolitis compared with age-matched healthy subjects. Sci Rep [Internet]. 2017;7:1–9. Available from: <http://dx.doi.org/10.1038/s41598-017-11311-z>

48. Salter SJ, Turner C, Watthanaworawit W, de Goffau MC, Wagner J, Parkhill J, et al. A longitudinal study of the infant nasopharyngeal microbiota: The effects of age, illness and antibiotic use in a cohort of South East Asian children. PLoS Negl Trop Dis [Internet]. 2017;11(10):1–17. Available from: https://journals.plos.org/plosntds/article?id=10.1371/journal.pntd.0005975

49. Carrión D, Kaali S, Kinney PL, Owusu-Agyei S, Chillrud S, Yawson AK, et al. Examining the relationship between household air pollution and infant microbial nasal carriage in a Ghanaian cohort. Environ Int [Internet]. 2019;133(April):105150. Available from: https://doi.org/10.1016/j.envint.2019.105150

50. Montgomery JM, Lehmann D, Smith T, Michael A, Joseph B, Lupiwa T, et al. Bacterial Colonization of the Upper Respiratory Tract and Its Association with Acute Lower Respiratory Tract Infections in Highland Children of Papua New Guinea. Rev Infect Dis [Internet]. 1990;12(8):S1006–16. Available from: https://doi.org/10.1093/clinids/12.Supplement_8.S1006

**Appendix 2**

**Characteristics of eligible studies**

|  | Study | Design | Age range (M) | Specimen(s) | Diagnostic test(s) | | Microbe(s) screened (N)^1^ | | | Population size (N) | Case group | | | | | | | Control group | | | | | | | Adjustment for confounders |
| --- | --- | --- | --- | --- | --- | --- | --- | --- | --- | --- | --- | --- | --- | --- | --- | --- | --- | --- | --- | --- | --- | --- | --- | --- | --- |
|  |  |  |  |  | Primary tests | Additional tests | Bacteria | Viruses | Fungi |  | Number of participants (%) | Definition (criteria) | Proportion male participants | Age (M) | Setting | Proportion of participants with antibiotic administration prior to sampling | Vaccination history (%) | Number of participants (%) | Definition (criteria) | Proportion male participants | Age (M) | Setting | Proportion of participants with antibiotic administration prior to sampling | Vaccination history (%) |  |
| 1 | PERCH Study Group 2019  (PERCH Study) | CSCC | 1-59 | NPS  OPS | Multiplex real time RT-qPCR using FTD Respiratory pathogens 33 kit  Culture | *S. pneumoniae* serotype analysis using Quellung reaction/PCR or microarray assay  *H. influenzae* serotype analysis using slide agglutination or PCR | 11 | 21 | 1 | 6721 | 1737 (26%) | SP & VSP (CXR; WHO guidelines) | HIV-CXR+ cases (N=1769): 56%* | HIV-CXR+ cases (N=1769):  1-5: 39%  6-11: 24%  12-23: 25%  24-59: 12% | H | CXR positive cases (N=1769): 40%* | CXR positive cases (n=1769):  DTP full vaccination includes Hib for 6/7 sites (<1 year: 70%*, >1 year: 91%)  PCV full vaccination (<1 year: 52%*, >1 year: 46%)  Measles full vaccination (85%)* | 4984 (74%) | Community controls [including those with URTI, or non-severe pneumonia symptoms (1% of control group)] | HIV- controls (N=5102): 50%* | HIV- controls (N=5102):  1-5: 31%  6-11: 24%  12-23: 25%  24-59: 20% | CO | All controls (N=5102): 2%* | All controls (N=5102):  DTP full vaccination iincludes Hib for 6/7 sites (<1 year: 81%*, >1 year: 94%)  PCV full vaccination (<1 year: 50%*, >1 year: 39%)  Measles full vaccination (91%) | Age |
| 2 | Park 2017  (PERCH Study) | CSCC | 1-59 | NPS  OPS | Multiplex real time RT-qPCR using FTD Respiratory pathogens 33 kit | - | 11 | 21 | 1 | 6643 | 1657 (25%)^2^ | SP & VSP (CXR; WHO guidelines) | HIV-CXR+ cases (N=1700): 57% | HIV-CXR+ cases (N=1700):  1-5: 40%^3^  6-11: 24%^3^  12-23: 24%^3^  24-59: 12%^3^ | H | HIV-CXR+ cases (N=1700): 47% | Entire cohort: Routine use of Hib and PCV administration differed across countries | 4986 (75%) | Community controls [including those with URTI, or non-severe pneumonia symptoms (1% of control group)] | HIV- controls (N=4986): 50% | HIV- controls (N=4986):  1-5: 31%  6-11: 24%  12-23: 25%  24-59: 20% | CO | HIV- controls (N=4986): 2% | Entire cohort: Routine use of Hib and PCV administration differed across countries | Age |
| 3 | Barger-Kamate 2016  (PERCH Study) | CSCC | 1-59 | NPS  OPS | Multiplex real time RT-qPCR using FTD Respiratory pathogens 33 kit | Samples positive for *Bordetella pertussis*: Uniplex *B. pertussis* IS*481* and *B. holmesii* *recA* PCR assays | 1 | 0 | 0 | 9396 | 4200 (45%) | SP & VSP (WHO guidelines) | NR | 1-5: 41%  6-59: 59% | H | NR | NR | 5196 (55%) | Community controls [including those with URTI, or non-severe pneumonia symptoms (1% of control group)] | NR | 1-5: 31%  6-59: 69% | CO | NR | NR | Age  Season |
| 4 | Baggett 2017  (PERCH Study) | CSCC | 1-59 | NPS  OPS | Multiplex real time RT-qPCR using FTD Respiratory pathogens 33 kit | *S. pneumoniae* serotype analysis using Quellung reaction or latex agglutination | 1 | 0 | 0 | 7997 | 4035 (51%)^2^ | SP & VSP (CXR; WHO guidelines) | 57% | 1-5: 41%  6-11: 23%  12-23: 22%  24-59: 14% | H | 46% | PCV (51%) | 3962 (49%) | No RI | 50% | 1-5: 33%  6-11: 23%  12-23: 24%  24-59: 20% | CO | 2% | PCV (50%) | Age  HIV status |
| 5 | Piralam 2020  (PERCH Study) | CSCC | 1-59 | NPS  OPS | Real time qPCR | *S. pneumoniae* serotype analysis using Quellung reaction | 1 | 0 | 0 | 872 | 222 (26%) | SP & VSP (CXR; WHO guidelines) | 60%* | 15 (7-26)†* | H | 65%* | PCV (1%) | 650 (74%) | Community controls [including those with URTI, or non-severe pneumonia symptoms (1% of control group)] | 51%* | 16 (8-33)^†^* | CO | 9%* | PCV (0%) | Age  Season |
| 6 | Camelo 2020  (PERCH Study) | CSCC | 1-59 | NPS  OPS | Real time qPCR | *S. pneumoniae* serotype analysis using Quellung reaction or latex agglutination | 1 | 0 | 1 | 1154 | 555 (48%) | SP & VSP (CXR; WHO guidelines) | 53% | 8 (±10)‡* | H | NR | NR | 599 (52%) | Community controls [including those with URTI, or non-severe pneumonia symptoms (1% of control group)] | 51% | 11 (±12)‡* | CO | NR | NR | Age  Season |
| 7 | Jroundi 2017 | CSCC | 2-59 | NPS | Culture | *S. pneumoniae* serotype analysis using multiplex real time PCR | 1 | 0 | 0 | 900 | 700 (78%) | SP (WHO guidelines) | 64% | 22 (±15)‡ | H | 29% | PCV13 at least 1 dose (17%) | 200 (22%) | Healthy controls (vaccinations) | (N=195): 49% | 9 (±11)‡ | PHC | (N=195): 17% | (N=195): PCV13 at least 1 dose (70%) | NR |
| 8 | PrayGod 2016 | CSCC | 2-59 | NPS | Culture | - | 5 | 0 | 0 | 117 | 45 (39%) | SP & VSP (WHO guidelines) | 51% | 16 (14)‡* | H | (N=32): 82%* | (N=41): Measles (54%)* | 72 (61%) | No ALRI | 54% | 26 (±16)‡* | H | (N=61): 40%* | (N=70): Measles (90%)* | Season |
| 9 | Kumar 2004 | CSCC | 2-60 | NPS | Culture | - | 4 | 0 | 0 | 100 | 50 (50%) | SP (WHO guidelines) | 70% | Entire cohort:  <12: 74%  12-60: 26% | H | 0% | Immunised (78%) | 50 (50%) | Healthy controls (vaccinations) | 70% | Entire cohort:  <12: 74%  12-60: 26% | O | 0% | Immunised (84%) | Age  Sex  Nutritional status |

**Appendix 2**

**Characteristics of eligible studies (continued)**

|  | Study | Design | Age range (M) | Specimen(s) | Diagnostic test(s) | | Microbe(s) screened (N)^1^ | | | Population size (N) | Case group | | | | | | | Control group | | | | | | | Adjustment for confounders |
| --- | --- | --- | --- | --- | --- | --- | --- | --- | --- | --- | --- | --- | --- | --- | --- | --- | --- | --- | --- | --- | --- | --- | --- | --- | --- |
|  |  |  |  |  | Primary tests | Additional tests | Bacteria | Viruses | Fungi |  | Number of participants (%) | Definition (criteria) | Proportion male participants | Age (M) | Setting | Proportion of participants with antibiotic administration prior to sampling | Vaccination history (%) | Number of participants (%) | Definition (criteria) | Proportion male participants | Age (M) | Setting | Proportion of participants with antibiotic administration prior to sampling | Vaccination history (%) |  |
| 10 | Hammitt 2012 | CSCC | 1-59 | NPS | Multiplex real time RT-qPCR | - | 1 | 15 | 0 | 947 | 805 (85%) | SP & VSP (WHO guidelines) | (N=810): 55%* | (N=810): 13φ*^3^ | H | NR | NR | 142 (15%) | No RI | (N=369): 45%* | (N=369): 20 φ*^3^ | O  PHC | NR | NR | Age  Season |
| 11 | Feikin 2013 | CSCC | 1-59 | NPS  OPS | Real time RT-qPCR | - | 1 | 10 | 0 | 292 | 199 (68%) | SARI (variation of WHO guidelines used for SP and VSP)  SARI from community (C, D, and CI or T) | NR | 18 (NR)† | H  CO | NR | NR | 93 (32%) | No RI (health care maintenance) | NR | 15 (NR)† | O | NR | NR | NR |
| 12 | Greenberg 2011 | CSCC | 0-60 | NPS | Culture | *S. pneumoniae* serotype analysis using Quellung reaction | 1 | 0 | 0 | 3015 | 960 (32%) | P (CXR as per WHO guidelines) | NR | NR | H | NR | PCV (0%) | 2055 (68%) | Controls (immunization clinics/elective surgery) | NR | NR | H | NR | PCV (0%) | NR |
| 13 | Adebanjo 2018 | CSCC | 0-59 | NPS | Culture  Culture negative swabs from cases: Real-time PCR targeting lytA gene | *S. pneumoniae* serotype analysis using Quellung reaction or conventional multiplex PCR | 1 | 0 | 0 | 1705 | 778 (46%) | P (CXR as per WHO guidelines) | 52% | 12 (7-18)†* | H | 97%* | PCV10 1or2 doses (17%)*  PCV10 3 doses (74%)* | 927 (54%) | No P | 52% | 15 (11-21)†* | O  CO | 27%* | PCV10 1or2 doses (10%)*  PCV10 3 doses (80%)* | NR |
| 14 | Ngocho 2020 | CSCC | 2-59 | NPS | Real time qPCR | *H. influenzae* multiplex PCR for the detection of capsule genes | 6 | 2 | 0 | 433 | 109 (25%) | P (CXR; WHO guidelines) | 61% | 14 (7-26)† | H | 7% | (N=107): PCV and Hib (87%)* | 324 (75%) | Healthy controls (no RI) | 58% | 15 (7-26)† | CO | (N=306): 13% | (N=323): PCV and Hib (94%)* | Age  Sex  Season |
| 15 | Rey 2002 | CSCC | 2-59 | NPS | Culture | *S. pneumoniae* antimicrobial susceptibility testing using MIC breakpoints  *S. pneumoniae* serotype analysis using antisera and Quellung reaction | 1 | 0 | 0 | 911 | 482 (53%) | P (WHO guidelines; no W) | NR | 2-11: 66%  12-59: 34% | ED | 32% | NR | 429 (47%) | No RI | NR | 2-11: 16%  12-59: 84% | PHC  CO | 7% | NR | NR |
| 16 | Wolf 2001 | CSCC | 2-60 | NPS | Culture | Antimicrobial susceptibility testing using Vitek-1 and/or agar diffusion tests | 21 | 0 | 0 | 912 | 482 (53%) | P (WHO guidelines; no W) | 54% | 20 (±13)‡* | ED | Entire cohort: 20% | PCV (0%)  Hib (0%) | 430 (47%) | Healthy controls (day-care centres and vaccination clinics) | 57% | 17 (±15)‡* | PHC  CO | Entire cohort: 20% | PCV (0%)  Hib (0%) | NR |
| 17 | Weber 2006 | CSCC | 3-60 | NPA | PCR | - | 1 | 0 | 0 | 325 | 208 (64%) | P (CXR) | NR | NR | O | NR | NR | 117 (36%) | No RI | NR | NR | O | NR | NR | NR |
| 18 | Benet 2015  (GABRIEL Network) | CSCC | 2-59 | NS | Multiplex real time RT-PCR using FTD Respiratory pathogens 21 plus kit | *S. pneumoniae* serotype analysis using multiplex real time PCR | 5 | 19 | 0 | 216 | 118 (55%) | P (C and/or D; T and CXR as per WHO guidelines; no W) | 48% | 12 (5-26)† | H | Yes | DTP-HepB-Hib-1 dose (86%)  DTP-HepB-Hib-3 dose (69%)  Influenza (0.8%) | 98 (45%) | No RI | 39% | 11 (5-23)† | H  O | NR | DPT-HepB-Hib-1 dose (86%)  DPT-HepB-Hib-3 dose (74%)  Influenza (0%) | Age  Season |
| 19 | Benet 2017  (GABRIEL Network) | CSCC | 2-59 | NPS | Multiplex real time RT-PCR using FTD Respiratory pathogens 21 plus kit | - | 5 | 19 | 0 | 1758 (1755 in results) | 888 (51%) | P (C and/or D; T and CXR as per WHO guidelines) | 59% | 2-11: 37%  12-23: 29%  24-60: 34% | H | 71% | PCV (3%)*  DTP-HepB-Hib 1 dose (78%)*  DTP-HepB-Hib 3 dose (68%)  Influenza (4%) | 870 (49%) | No RI | 57% | 2-11: 34%  12-23: 27%  24-60: 39% | H  O | NR | PCV (9%)*  DPT-HepB-Hib 1 dose (87%)*  DPT-HepB-Hib 3 dose (72%)  Influenza (5%) | Age  Site  Period of admission |
| 20 | Dananche 2019  (GABRIEL Network) | CSCC | 2-60 | NPS | Multiplex real time RT-PCR using FTD Respiratory pathogens 21 plus kit | *S. pneumoniae* serotype analysis using multiplex real time PCR | 5 | 19 | 0 | 1758 | 888 (51%) | P (C and/or D, T, and CXR as per WHO guidelines) | 59% | 20 (9-28)† | H | 63% | PCV (3%) | 870 (49%) | No RI (hospitalized for surgery / attending routine outpatient appointment) | 57% | 17 (9-33)† | H  O | 28% | PCV (9%) | Age  Season  *Subset of participants* |

**Appendix 2**

**Characteristics of eligible studies (continued)**

|  | Study | Design | Age range (M) | Specimen(s) | Diagnostic test(s) | | Microbe(s) screened (N)^1^ | | | Population size (N) | Case group | | | | | | | Control group | | | | | | | Adjustment for confounders |
| --- | --- | --- | --- | --- | --- | --- | --- | --- | --- | --- | --- | --- | --- | --- | --- | --- | --- | --- | --- | --- | --- | --- | --- | --- | --- |
|  |  |  |  |  | Primary tests | Additional tests | Bacteria | Viruses | Fungi |  | Number of participants (%) | Definition (criteria) | Proportion male participants | Age (M) | Setting | Proportion of participants with antibiotic administration prior to sampling | Vaccination history (%) | Number of participants (%) | Definition (criteria) | Proportion male participants | Age (M) | Setting | Proportion of participants with antibiotic administration prior to sampling | Vaccination history (%) |  |
| 21 | Zar 2016 | CSCC | NR  (0-24) | NPS | Multiplex real time RT-qPCR using FTD Respiratory pathogens 33 kit | - | 11 | 21 | 1 | 702 | 284 (41%) | P (WHO guidelines) | 68%* | 5 (3-9)† | H  PHC | 84% | (N=283): PCV13 + DTaP-IPV-Hib 6Wks (99% of which 13% delayed 2Wks)  (N=275): DTaP-IPV-Hib 10Wks (98% of which 21% delayed 2Wks)  (N=274): PCV13 + DTaP-IPV-Hib 14Wks (95% of which 33% delayed 2Wks)  (N=200): PCV13 + Measles 9M (97% of which 23% delayed 2Wks) | 418 (59%) | No P (+/- URTI) | 55%* | 5 (2-8)† | PHC | NR | (N=415): PCV13 + DTaP-IPV-Hib 6Wks (100% of which 8% delayed 2Wks)  (N=411): DTaP-IPV-Hib 10Wks (100% of which 17% delayed 2Wks)  (N=408): PCV13 + DTaP-IPV-Hib 14Wks (97% of which 25% delayed 2Wks)  (N=334): PCV13 + Measles 9M (94% of which 19% delayed 2Wks) | Age  Site |
| 22 | Kelly 2017 | CSCC | 1-23 | NPS | 16S rRNA gene sequencing (hypervariable region: V3)  Real time qPCR | - | Community-wide profile + 1 | 15 | 0 | 264 | 204 (77%) | P (WHO guidelines) | 50% | 8 (±6)‡ | ED | 22%* | PCV13 0 doses (59%)*  PCV13 1 dose (9%)*  PCV13 >2 doses (31%)* | 60 (23%) | No RI | 40% | 8 (±5)‡ | PHC | 0%* | PCV13 0 doses (50%)*  PCV13 1 dose (12%)*  PCV13 >2 doses (38%)* | Season  Site |
| 23 | Levine 2000 | CSCC | 2-60 | NPS | Culture | *S. pneumoniae* and *H. influenzae* serotype analysis using antisera | 2 | 0 | 0 | 310 | 96 (31%) | P (CXR and at least 3 of the following: C, T, F, CI, AF) | 52% | 2-5: 21%  6-11: 9%  12-35: 43%  36-60: 27% | O | 60%* | Hib (0%) | 214 (69%) | No RI | 61% | 2-5: 19%  6-11: 18%  12-35: 39%  36-60: 24% | O | 32%* | Hib (0%) | Age |
| 24 | Sutcliffe 2019 | CSCC | 2-59 | NPS | Culture | *S. pneumoniae* serotype analysis using Quellung reaction and PCR  Antimicrobial susceptibility testing using MIC breakpoints | 1 | 0 | 0 | 601 | 91 (15%) | P (F and T or >1 danger signs for P, excluded if symptoms were consistent with acute B or suspicion of viral infection) | 59% | 2-11: 25%  12-23: 30%  24-59: 45% | H  PHC | 20% | Prior to PCV13 introduction | 510 (85%) | Community controls without RI (PHC, immunization clinics, day care centres) | 50% | 2-11: 26%  12-23: 22%  24-59: 53% | PHC  CO | NR | Prior to PCV13 introduction | NR |
| 25 | Mogdasy 1992 | CSCC | 1-60 | TS | Culture | - | 1 | 0 | 0 | 651 | 344 (53%) | P (clinical symptoms including pulmonary consolidation) | NR | 0-5: 24%  6-11: 28%  12-23: 24%  24-60: 24% | H | NR | NR | 307 (47%) | Healthy controls | NR | 0-5: 26%  6-11: 24%  12-23: 27%  24-60: 23% | PHC | NR | NR | Age  Season |
| 26 | Macasaet 1968 | CSCC | 0-60 | OPS  NS | Culture | - | 4 | 10 | 0 | 106 | 48 (45%) | P (clinical diagnosis, CXR) | NR | 0-6: 23%  7-12: 17%  13-24: 42%  25-60: 19% | H | 0% | NR | 58 (55%) | No RI | NR | 0-6: 29%  7-12: 19%  13-24: 29%  25-60: 22% | H | 0% | NR | NR |
| 27 | Bhuiyan 2019 | CSCC | 0-12 | NPS | Multiplex real time qPCR | - | 6 | 14 | 0 | 38 | 15 (40%) | P (CXR and RI+/-F) | NR | NR | H | NR | At population level: >90% | 23 (60%) | Healthy controls (no RI) | NR | NR | O  PHC | NR | At population level: >90% | Age |
| 28 | Liu 2005 | CSCC | 2-60 | NPS | Real time PCR | - | 2 | 0 | 0 | 270 | 85 (32%) | P (CXR and at least 3 of the following: C, T, F, CI, AF) | 52% | 2-5: 18%  6-11: 9%  12-35: 47%  36-60: 26% | O | 84%* | NR | 185 (68%) | No RI | 62% | 2-5: 16%  6-11: 20%  12-35: 41%  36-60: 23% | O | 61%* | NR | Age |
| 29 | Palmu 2019 | LCC | 0-24 | NS | Multiplex real time RT-qPCR | - | 8 | 17 | 0 | 151 (8100 specimen) | NR | LRTI (parental report of C, D, RB, W, or doctor-diagnosed P) | NR | NR | CO | (N=158): 57% | PCV7 or PCV13: all cohort subjects completed primary PCV series. Overall coverage: 152/158 (96%) | NR | All swabs (asymptomatic, LRTI, URTI and AOM) | NR | NR | CO | NR | PCV7 or PCV13: all cohort subjects completed primary PCV series. Overall coverage: 152/158 (96%) | NR |

**Appendix 2**

**Characteristics of eligible studies (continued)**

|  | Study | Design | Age range (M) | Specimen(s) | Diagnostic test(s) | | Microbe(s) screened (N)^1^ | | | Population size (N) | Case group | | | | | | | Control group | | | | | | | Adjustment for confounders |
| --- | --- | --- | --- | --- | --- | --- | --- | --- | --- | --- | --- | --- | --- | --- | --- | --- | --- | --- | --- | --- | --- | --- | --- | --- | --- |
|  |  |  |  |  | Primary tests | Additional tests | Bacteria | Viruses | Fungi |  | Number of participants (%) | Definition (criteria) | Proportion male participants | Age (M) | Setting | Proportion of participants with antibiotic administration prior to sampling | Vaccination history (%) | Number of participants (%) | Definition (criteria) | Proportion male participants | Age (M) | Setting | Proportion of participants with antibiotic administration prior to sampling | Vaccination history (%) |  |
| 30 | Anh 2007 | CSCC | 0-59 | NPS | Culture | - | 7 | 0 | 0 | 234 | 164 (70%) | LRTI (C, T, F and AF) with further classification as P or B (CXR as per WHO guidelines) | P group: 63%  B group: 73% | P group: 15 (±12)‡  B group: 12 (±9)‡ | H | 70% | PCV (0%)  Hib (0%) | 70 (30%) | Healthy controls (day-care centres) | 51% | 15 (±9)‡ | CO | NR | PCV (0%)  Hib (0%) | NR |
| 31 | Bhuyan 2017 | CSCC | 0-59 | NS | Culture for bacteria  Singleplex real time RT-qPCR | Antimicrobial susceptibility testing using MIC breakpoints | 5 | 10 | 0 | 230 | 200 (87%) | ARI (clinical symptoms and/or CXR suggestive of pneumonia; no chronic RI) | 62% | 10 (NR)‡ | H | Not during hospitalisation | NR | 30 (13%) | No RI | NR | 13 (NR)‡ | H | Not during hospitalisation | NR | NR |
| 32 | Mastro 1993 | CSCC | 1-60 | TS | Culture | *S. pneumoniae* and *H. influenzae* serotype analysis using antisera  Antimicrobial susceptibility testing using agar dilution method | 2 | 0 | 0 | 734^4^ | 601 (82%)^4^ | ARI (C/D and CI/T/F, no W) | 61%^4^ | 15 (NR)‡*^4^ | O  ED | NR | NR | 133 (18%)^4^ | Healthy controls without RI (immunization clinics) | 56%^4^ | 11 (NR)‡* ^4^ | PHC | NR | NR | NR |
| 33 | Vathanophas 1990 | CSCC | 0-60 | TS  NPA | Culture | Antimicrobial susceptibility tests | 3 | 9 | 0 | 326 (1305 specimens) | 10 LRTI specimens (1%) | LRTI (W, T, CY, AF or RB) | NR | NR | CO | NR | NR | 1295 healthy and mild URTI specimens (99%) | Healthy and mild URTI controls (no LRTI) | NR | NR | CO | NR | NR | NR |
| 34 | Smith-Vaughan 2018 | LCC (SCC group); CSCC (DCC group) | 3-24 | NPS | Real time qPCR  Real time RT-qPCR | Human adenovirus and Human rhinovirus typing | 6 | 18 | 0 | SCC group: 232 specimens  DCC group: 325 specimens | SCC group: 75 (120 specimens - 52%)  DCC group: 101 (170 specimens - 52%) | Hospitalized LRTI (F and at least 1 of the following: CXR, T, CR/W/AF)  Community LRTI (F and CI/T ± CR/W)  Sub-diagnosis of P and B (medical records) | SCC group: 66%  DCC group: 64%* | SCC group: 7 (4-21)†*  DCC group: 7 (3-21)† | H  PHC | SCC group: 11%*  DCC group: 11%* | SCC group: >2 doses PCV7 (35%)*  DCC group: >2 doses PCV7 (30%) | SCC group: 75 (112 specimens – 48%)  DCC group: 86 (155 specimens – 48%) | SCC controls: 90-180 prior to LRTI episode, or 25-90 days prior to LRTI if episode occurred at <180 days of age  DCC controls: No LRTIs within 21 days of collection | SCC group: 64%  DCC group: 52%* | SCC group: 5 (3-16)†*  DCC group: 7 (3-20)† | H  PHC | SCC group: 17%*  DCC group: 19%* | SCC group: >2 doses PCV7 (17%)*  DCC group: >2 doses PCV7 (31%) | DCC group:  Age  Season |
| 35 | Coles 2009 | CSCC | 1-36 | NPS | Culture | *S. pneumoniae* antimicrobial susceptibility testing using MIC breakpoints  *S. pneumoniae* serotype analysis using antisera | 1 | 0 | 0 | 1100 | 550 (50%) | LRTI (C, F and T/D/CI) | 49% | 1-11: 27%  12-36: 73% | CO | NR | NR | 550 (50%) | No LRTI | 50% | 1-11: 27%  12-36: 73% | CO | NR | NR | Age  Season |
| 36 | Vu 2011 | CSCC | 0-59 | NPS | Multiplex real time RT-qPCR | *S. pneumoniae* serotype analysis using multiplex real time PCR | 3 | 13 | 0 | 900 | 550 (61%) | LRTI (C and/or D and abnormal CXR)  P (subset of LRTI cases with CXR as per WHO guidelines) | LRTI group: 60%  P group: 61%* | LRTI group: 18 (12-25)†*  P group: 16 (9-24)†* | H | LRTI group: 33%  P group: 36% | NR | 350 (39%) | No RI | 53%* | 20 (9-39)†* | CO | 0% | NR | NR |
| 37 | Bezerra 2011 | CSCC | 0-60 | NPA | Multiplex real time RT-qPCR | - | 2 | 8 | 0 | 343 | 320 (93%) | B (clinical diagnosis: URTI preceding W,T)  P (clinical diagnosis: F,T,CR/AF +/- CXR) | NR | NR | H  ED | NR | NR | 23 (7%) | URTI (clinical diagnosis: no ALRI) | NR | NR | ED | NR | NR | NR |

**Appendix 2**

**Characteristics of eligible studies (continued)**

|  | Study | Design | Age range (M) | Specimen(s) | Diagnostic test(s) | | Microbe(s) screened (N)^1^ | | | Population size (N) | Case group | | | | | | | Control group | | | | | | | Adjustment for confounders |
| --- | --- | --- | --- | --- | --- | --- | --- | --- | --- | --- | --- | --- | --- | --- | --- | --- | --- | --- | --- | --- | --- | --- | --- | --- | --- |
|  |  |  |  |  | Primary tests | Additional tests | Bacteria | Viruses | Fungi |  | Number of participants (%) | Definition (criteria) | Proportion male participants | Age (M) | Setting | Proportion of participants with antibiotic administration prior to sampling | Vaccination history (%) | Number of participants (%) | Definition (criteria) | Proportion male participants | Age (M) | Setting | Proportion of participants with antibiotic administration prior to sampling | Vaccination history (%) |  |
| 38 | Suarez-Arrabal 2015 | CSCC | 0-24 | NPS  NW | Culture | - | 7 | 0 | 0 | 95 | 72 (76%) | RSV-B (confirmatory RSV tests) | 63% | 3 (2-4)† | H | 0% | Up to date vaccination status (85%)^5^ | 23 (24%) | No RI | 65% | 3 (2-8)† | H  PHC | 0% | Up to date vaccination status (87%) | Age |
| 39 | Kumar 2005 | CSCC | 0-18 | NPS | Culture  PCR for *Simkania negevensis* | - | 2 | 0 | 0 | 100 | 66 (66%) | B (NR) | NR | NR | H  O  ED | NR | NR | 34 (34%) | Healthy controls (health care maintenance) | NR | NR | O | NR | NR | Age |
| 40 | Kahane 1998 | CSCC | 0.5-12 | NPS  NW | PCR | - | 1 | 3 | 0 | 317 | 239 (75%) | B (T, D, W and hyperinflation ± atelectasis or CXR infiltrates) | NR | NR | ED | NR | NR | 78 (25%) | No RI | NR | NR | H | NR | NR | Age  Season |
| 41 | Man 2019 | CSCC | 1-60 | NPS | 16S rRNA gene sequencing (hypervariable region: V4)  Multiplex real time PCR (RespiFinder SMARTfast 22)  qPCR | - | Community-wide profiles + 4 | 18 | 0 | 457 | 151 (33%) | LRTI (WHO guidelines; required mechanical ventilation)  LRTI phenotypes: P, B, W illness, mixed | (N=154): 60% | (N=154): 14 (5-27)† | H | (N=154): 27%* | NR | 306 (67%) | Healthy controls | (N=307): 60% | (N=307): 14 (5-28)† | PHC  CO | (N=307): 6%* | NR | Age  Sex  Season |
| 42 | Teo 2015  CAS Study | LCC | 0-12 | NPA | 16S rRNA gene sequencing (hypervariable region: V4)  Multiplex real time RT-qPCR | - | Community-wide profile | 14 | 0 | 234 (1021 specimens) | 380 LRTI-state specimens (viral PCR: 327 specimens) | LRTI (RI with parental report of W) | 56% | NR | CO | NR | NR | 487 healthy-state specimens (viral PCR: 451)  154 URTI-state specimens (viral PCR: 649) | Healthy: No RI for at least 4 Wks prior collection  URTI: RI, no parental reported W | 56% | NR | CO | NR | NR | NR |
| 43 | Teo 2018  CAS Study | LCC | 0-60 | NPA | 16S rRNA gene sequencing (hypervariable region: V4)  Multiplex real time RT-qPCR | - | Community-wide profile | 14 | 0 | 244 (3014 specimens) | 1032 LRTI-state specimens (viral PCR: 789 specimens) | LRTI (RI with parental report of W) | 57% | NR | CO | NR | NR | 1018 healthy-state specimens (viral PCR: 736)  964 URTI-state specimens (viral PCR: 583) | Healthy: No RI for at least 4 Wks prior collection  URTI: RI, no parental reported W | 57% | NR | CO | NR | NR | NR |
| 44 | De Steenhuijsen Piters 2016 | CSCC | 0-24 | NPS | 16S rRNA gene sequencing (hypervariable region: V5-V7)  Real time qPCR | - | Community-wide profiles + 4 | 0 | 0 | 110 | 84 (76%) | RSV infection (hospitalised, clinical disease severity score: mild 21%, moderate 49%, severe 30%) | 60% | 3 (1-5)†* | H | 46%* | NR | 26 (24%) | Controls (PHC/elective surgery, no RI) | 73% | 7 (2-10)†* | H  PHC | 0%* | NR | NR |
| 45 | Hasegawa 2017 | CSCC | 0-12 | NS | 16S rRNA gene sequencing (hypervariable region: V4) | - | Community-wide profile | 0 | 0 | 150 | 40 (27%) | B (physician diagnosed: ARI with combinations of C, T, W, R, CR and/or RTR) | 55% | 4 (±3)‡ | H | 21% | NR | 110 (73%) | No RI | 56% | 4 (±2)‡ | PHC | 13% | NR | Age |

**Appendix 2**

**Characteristics of eligible studies (continued)**

|  | Study | Design | Age range (M) | Specimen(s) | Diagnostic test(s) | | Microbe(s) screened (N)^1^ | | | Population size (N) | Case group | | | | | | | Control group | | | | | | | Adjustment for confounders |
| --- | --- | --- | --- | --- | --- | --- | --- | --- | --- | --- | --- | --- | --- | --- | --- | --- | --- | --- | --- | --- | --- | --- | --- | --- | --- |
|  |  |  |  |  | Primary tests | Additional tests | Bacteria | Viruses | Fungi |  | Number of participants (%) | Definition (criteria) | Proportion male participants | Age (M) | Setting | Proportion of participants with antibiotic administration prior to sampling | Vaccination history (%) | Number of participants (%) | Definition (criteria) | Proportion male participants | Age (M) | Setting | Proportion of participants with antibiotic administration prior to sampling | Vaccination history (%) |  |
| 46 | Ederveen 2018 | CSCC | 0-6 | NPA | 16S rRNA gene sequencing (hypervariable region: V3-V4)  Multiplex real time RT-qPCR | IL-6, CXCL8, CXCL10, CCL5, and MMP9 levels were measured by ELISA | Community-wide profiles + 4 | 15 | 0 | 75 | 54 (72%) | RSV-B: mild, moderate, severe (confirmatory RSV tests) | 50% | 2 (1-3)† | H | 11% | NR | 21 (28%) | Healthy (elective surgery): No RI, no RSV | 24% | 3 (2-4)† | H | 0% | NR | Age |
| 47 | Hu 2017 | CSCC | 0-12 | OPS | 16S rRNA gene sequencing (hypervariable region: V3-V4) | - | Community-wide profiles + 4 | 0 | 0 | 49 | 27 (55%) | RSV-B: mild (nebulised) | 67% | 4 (1-11)† | H | 0% | NR | 22 (45%) | No RI | 32% | 6 (1-11)† | CO | 0% | NR | Age |
| 48 | Salter 2017 | LCC | 0-24 | NPS  NPA | 16S rRNA gene sequencing (hypervariable region: V3-V4)  Culture  Real time RT-qPCR for viruses | *S. pneumoniae* serotype analysis using latex agglutination | Community-wide profiles + 4 | 4 | 0 | 21 (517 specimens) | 47 LRTI-state specimens (9%) | P (CXR as per WHO guidelines) | 24% | NR | PHC | During study period: 86% of cohort | PCV (0%)  Hib (0%) | 470 healthy-state specimens (91%) | No P | 24% | NR | PHC | During study period: 86% of cohort | PCV (0%)  Hib (0%) | NR |
| 49 | Carrion 2019 | CSCC | 0-12 | NPS | MassTag PCR | - | 9 | 13 | 0 | 260 | 130 (50%) | P (WHO guidelines, clinically diagnosed) | 52% | Intention to treat group: 5 (±3)‡  Exposures response group: 5 (±3)‡ | CO | NR | PCV 3 doses: 91% of cohort subjects | 130 (50%) | Healthy controls | 55% | Intention to treat group: 6 (±3)‡  Exposure response group: 6 (±3)‡ | CO | NR | PCV 3 doses: 91% of cohort subjects | Age  Sex  Site |
| 50 | Montgomery 1990 | LCC | 0-60 | NS | Culture | *S. pneumoniae* and *H. influenzae* serotype analysis using antisera  Antimicrobial susceptibility testing using agar dilution method | 22 | 0 | 0 | 150 | 72 (48%) | ALRI (C and T, ± CI) | NR | NR | CO | NR | NR | 78 (52%) | No ALRI | NR | NR | CO | NR | NR | NR |

CAS - Childhood Asthma Study; GABRIEL - Global Approach to Biological Research, Infectious diseases and Epidemics in Low-income countries; PERCH - Pneumonia Etiology Research for Child Health;

CSCC - Cross-Sectional Case-Control; LCC - Longitudinal Case-Control; DCC - Different Child Control cohort; SCC - Same Child Control cohort

NPA - Nasopharyngeal aspirates; NPS - Nasopharyngeal swabs; NS - Nasal swabs; NW - Nasal wash; OPS - Oropharyngeal swab

FTD - Fast Track Diagnostic Respiratory Pathogens 21 Kit; MIC - Minimum inhibitory concentration; lytA - Autolysin-Encoding Gene; PCR - Polymerase Chain Reaction (RT - Reverse Transcription; q - Quantitative); rRNA - Ribosomal Ribonucleic Acid

AF - Auscultation findings indicative of lower respiratory disease; AOM - Acute Otitis Media; AS - Asymptomatic; C - Cough; CI - Chest Indrawing; CR - Crackles; CXR - Chest Radiography; CY - Cyanosis; D - Dyspnea; F - Fever; LRTI - Lower Respiratory Tract Infection; MCPP - Microbiologically Confirmed Pneumococcal Pneumonia; R - Rhinitis; RB - Rattly Breathing; RI - Respiratory Illness; RTR - Retractions; T - Tachypnea; URTI - Upper Respiratory Tract Infection; W - Wheeze; WHO - World Health Organisation; (RSV)B - (RSV - Respiratory Syncytial Virus) Bronchiolitis; (S)ARI - (Severe) Acute Respiratory Infection; (S/VS) P - (Severe/Very Severe) Pneumonia

CO - Community; ED - Emergency Department; H - Hospital; O - Outpatient; PHC - Primary Healthcare Clinics (including immunisation and study clinics)

DTaP-IPV-Hib - Diphtheria, Tetanus, acellular Pertussis, inactivated Polio Vaccine, *Haemophilus influenza* type b; DTP-HepB-Hib - Diphtheria, Tetanus, Pertussis, Hepatitis B, *Haemophilus influenza* type b; PCV - Pneumococcal conjugate vaccine

M - month(s); NR - Not reported; Wks - week(s)

^1.^Microbes screened for which results were provided from both cases and controls are listed in the sections below

^2.^Chest radiography (CXR)+ cases

^3.^Data provided is based on all participants enrolled in case- or control-groups

^4.^Children enrolled from urban setting

^5.^Vaccination status reported for all cases with and without prior antibiotic use (n=136)

*Significant difference reported when comparing cases and controls

^†^Median (interquartile range)

^‡^Mean (±standard deviation)

^φ^Average

**Appendix 2**

**Global distribution of studies**

**Single-centre studies**

| Income category | Country | Study number | Study details | Number of participants | Included in quantitative analyses (N=42) |
| --- | --- | --- | --- | --- | --- |
| High-income | Australia | 27 | Bhuiyan 2019 | 38 | ✓ |
|  |  | 29 | Palmu 2019 | 151 | ✓ |
|  |  | 34 | Smith-Vaughan 2018 | 104 | ✓ |
|  |  | 42 | Teo 2015 | 234 | 🗶 |
|  |  | 43 | Teo 2018 | 244 | ✓ |
|  | Israel | 12 | Greenberg 2011 | 3015 | ✓ |
|  |  | 40 | Kahane 1998 | 317 | ✓ |
|  | Netherlands | 41 | Man 2019 | 457 | ✓ |
|  |  | 46 | Ederveen 2018 | 75 | ✓ |
|  | United States of America | 26 | Macasaet 1968 | 106 | ✓ |
|  |  | 38 | Suarez-Arrabal 2015 | 95 | ✓ |
|  |  | 39 | Kumar 2005 | 100 | ✓ |
|  |  | 44 | De Steenhuijsen Piters 2016 | 110 | ✓ |
|  | Uruguay | 25 | Mogdasy 1992 | 651 | ✓ |
| Upper-middle-income | Botswana | 22 | Kelly 2017 | 264 | ✓ |
|  | Brazil | 15 | Rey 2002 | 911 | ✓ |
|  |  | 16 | Wolf 2001 | 912 | ✓ |
|  |  | 37 | Bezerra 2011 | 343 | ✓ |
|  | China | 23 | Levine 2000 | 310 | ✓ |
|  |  | 28 | Liu 2005 | 270 | ✓ |
|  |  | 47 | Hu 2017 | 49 | ✓ |
|  | Thailand | 33 | Vathanophas 1990 | 326 | ✓ |
|  |  | 48 | Salter 2017 | 21 | ✓ |
| Lower-middle-income | Bangladesh | 31 | Bhuyan 2017 | 230 | ✓ |
|  | Ghana | 49 | Carriὀn 2019 | 260 | 🗶 |
|  | India | 9 | Kumar 2004 | 100 | ✓ |
|  |  | 24 | Sutcliffe 2019 | 601 | ✓ |
|  | Kenya | 10 | Hammitt 2012 | 947 | ✓ |
|  |  | 11 | Feikin 2013 | 292 | ✓ |
|  | Morocco | 7 | Jroundi 2016 | 900 | ✓ |
|  | Pakistan | 32 | Mastro 1993 | 734 | ✓ |
|  | Papua New Guinea | 50 | Montgomery 1990 | 150 | ✓ |
|  | Tanzania | 8 | PrayGod 2016 | 117 | ✓ |
|  |  | 14 | Ngocho 2020 | 433 | ✓ |
|  | Vietnam | 30 | Anh 2007 | 234 | ✓ |
|  |  | 36 | Vu 2011 | 900 | ✓ |
|  | Zambia | 6 | Camelo 2020 | 1154 | 🗶 |
| Low-income | Mali | 18 | Bénet 2015 | 216 | ✓ |
|  | Nepal | 35 | Coles 2009 | 1100 | ✓ |
|  | The Gambia | 17 | Weber 2006 | 325 | ✓ |

**Appendix 2**

**Global distribution of studies (continued)**

**Multi-centre studies**

| Income category | Country | Study number | Study details | Number of participants | Included in quantitative analyses (N=42) |
| --- | --- | --- | --- | --- | --- |
| High-income | United States of America | 45 | Hasegawa 2017 | 150 | ✓ |
| Upper-middle-income | China | 19 | Bénet 2017 | 177 | ✓ |
|  |  | 20 | Dananché 2019 | 177 | 🗶 |
|  | Paraguay | 19 | Bénet 2017 | 199 | ✓ |
|  |  | 20 | Dananché 2019 | 199 | 🗶 |
|  | South Africa | 1 | PERCH Study Group 2019 | 1256 | ✓ |
|  |  | 2 | Park 2017 | 1242 | 🗶 |
|  |  | 3 | Barger-Kamate 2016 | 1876 | 🗶 |
|  |  | 4 | Baggett 2017 | 1814 | 🗶 |
|  |  | 21 | Zar 2016 | 702 | ✓ |
|  | Thailand | 1 | PERCH Study Group 2019 | 755 | ✓ |
|  |  | 2 | Park 2017 | 753 | 🗶 |
|  |  | 3 | Barger-Kamate 2016 | 880 | 🗶 |
|  |  | 4 | Baggett 2017 | 622 | 🗶 |
|  |  | 5 | Piralam 2020 | 872 | 🗶 |
| Lower-middle-income | Bangladesh | 1 | PERCH Study Group 2019 | 990 | ✓ |
|  |  | 2 | Park 2017 | 985 | 🗶 |
|  |  | 3 | Barger-Kamate 2016 | 1293 | 🗶 |
|  |  | 4 | Baggett 2017 | 1098 | 🗶 |
|  | Cambodia | 19 | Bénet 2017 | 271 | ✓ |
|  |  | 20 | Dananché 2019 | 272 | 🗶 |
|  | India | 19 | Bénet 2017 | 333 | ✓ |
|  |  | 20 | Dananché 2019 | 333 | 🗶 |
|  | Kenya | 1 | PERCH Study Group 2019 | 1137 | ✓ |
|  |  | 2 | Park 2017 | 1127 | 🗶 |
|  |  | 3 | Barger-Kamate 2016 | 1489 | 🗶 |
|  |  | 4 | Baggett 2017 | 1272 | 🗶 |
|  | Mongolia | 19 | Bénet 2017 | 201 | ✓ |
|  |  | 20 | Dananché 2019 | 201 | 🗶 |
|  | Zambia | 1 | PERCH Study Group 2019 | 723 | ✓ |
|  |  | 2 | Park 2017 | 715 | 🗶 |
|  |  | 3 | Barger-Kamate 2016 | 1213 | 🗶 |
|  |  | 4 | Baggett 2017 | 1059 | 🗶 |

PERCH - Pneumonia Etiology Research for Child Health

**Appendix 2**

**Global distribution of studies (continued)**

**Multi-centre studies**

| Income category | Country | Study number | Study details | Number of participants | Included in quantitative analyses (N=42) |
| --- | --- | --- | --- | --- | --- |
| Low-income | Haiti | 19 | Bénet 2017 | 223 | ✓ |
|  |  | 20 | Dananché 2019 | 223 | 🗶 |
|  | Madagascar | 19 | Bénet 2017 | 140 | ✓ |
|  |  | 20 | Dananché 2019 | 142 | 🗶 |
|  | Mali | 1 | PERCH Study Group 2019 | 963 | ✓ |
|  |  | 2 | Park 2017 | 945 | 🗶 |
|  |  | 3 | Barger-Kamate 2016 | 1397 | 🗶 |
|  |  | 4 | Baggett 2017 | 1073 | 🗶 |
|  |  | 19 | Bénet 2017 | 211 | ✓ |
|  |  | 20 | Dananché 2019 | 211 | 🗶 |
|  | Mozambique | 13 | Adebanjo 2018 | 1705 | ✓ |
|  | The Gambia | 1 | PERCH Study Group 2019 | 897 | ✓ |
|  |  | 2 | Park 2017 | 876 | 🗶 |
|  |  | 3 | Barger-Kamate 2016 | 1248 | 🗶 |
|  |  | 4 | Baggett 2017 | 1059 | 🗶 |

PERCH - Pneumonia Etiology Research for Child Health

**Appendix 2**

**Quality assessment for cross-sectional and prospective case-control studies**

| Study number | Study details | Selection | | | | Comparability | Outcome | | | Score | Risk of bias |
| --- | --- | --- | --- | --- | --- | --- | --- | --- | --- | --- | --- |
|  |  | Case definition adequate | Representativeness of the cases | Selection of Controls | Definition of Controls | Comparability of cases and controls based on the design or analysis | Ascertainment of exposure | Same method of ascertainment for cases and controls | Non-Response rate |  |  |
| 1 | PERCH Study Group 2019 | 🟉 | 🟉 | 🟉 | 🟉 | 🟉🟉 | 🟉 | 🟉 | 🟉 | 9 | LOW |
| 2 | Park 2007 | 🟉 | 🟉 | 🟉 | 🟉 | 🟉🟉 | 🟉 | 🟉 | 🟉 | 9 | LOW |
| 3 | Barger-Kamate 2006 | 🟉 | 🟉 | 🟉 | 🟉 | 🟉🟉 | 🟉 | 🟉 | 🟉 | 9 | LOW |
| 4 | Baggett 2017 | 🟉 | 🟉 | 🟉 | 🟉 | 🟉🟉 | 🟉 | 🟉 | 🟉 | 9 | LOW |
| 5 | Piralam 2020 | 🟉 | - | 🟉 | 🟉 | 🟉🟉 | 🟉 | 🟉 | 🟉 | 8 | LOW |
| 6 | Camelo 2020 | 🟉 | - | 🟉 | 🟉 | 🟉🟉 | 🟉 | 🟉 | - | 7 | LOW |
| 7 | Jroundi 2017 | 🟉 | 🟉 | 🟉 | 🟉 | - | 🟉 | 🟉 | 🟉 | 7 | LOW |
| 8 | PrayGod 2016 | 🟉 | - | - | 🟉 | 🟉 | 🟉 | 🟉 | - | 5 | HIGH |
| 9 | Kumar 2004 | 🟉 | - | 🟉 | 🟉 | 🟉🟉 | 🟉 | 🟉 | 🟉 | 8 | LOW |
| 10 | Hammitt 2012 | 🟉 | 🟉 | 🟉 | 🟉 | 🟉🟉 | 🟉 | 🟉 | 🟉 | 9 | LOW |
| 11 | Feikin 2013 | 🟉 | - | 🟉 | 🟉 | - | 🟉 | 🟉 | - | 5 | HIGH |
| 12 | Greenberg 2011 | 🟉 | - | 🟉 | - | - | 🟉 | 🟉 | 🟉 | 5 | HIGH |
| 13 | Adebanjo 2018 | 🟉 | 🟉 | 🟉 | 🟉 | 🟉🟉 | 🟉 | - | 🟉 | 8 | LOW |
| 14 | Ngocho 2020 | 🟉 | - | 🟉 | 🟉 | 🟉🟉 | 🟉 | 🟉 | 🟉 | 8 | LOW |
| 15 | Rey 2002 | 🟉 | 🟉 | 🟉 | 🟉 | - | 🟉 | 🟉 | 🟉 | 7 | LOW |
| 16 | Wolf 2001 | 🟉 | 🟉 | 🟉 | 🟉 | - | 🟉 | 🟉 | 🟉 | 7 | LOW |
| 17 | Weber 2006 | 🟉 | - | - | 🟉 | - | 🟉 | 🟉 | 🟉 | 5 | HIGH |
| 18 | Benet 2015 | 🟉 | - | - | 🟉 | 🟉🟉 | 🟉 | 🟉 | 🟉 | 7 | LOW |
| 19 | Benet 2017 | 🟉 | 🟉 | - | 🟉 | 🟉🟉 | 🟉 | 🟉 | 🟉 | 8 | LOW |
| 20 | Dananche 2019 | 🟉 | 🟉 | - | 🟉 | 🟉🟉 | 🟉 | 🟉 | 🟉 | 8 | LOW |

**Appendix 2**

**Quality assessment for cross-sectional and prospective case-control studies (continued)**

| Study number | Study details | Selection | | | | Comparability | Outcome | | | Score | Risk of bias |
| --- | --- | --- | --- | --- | --- | --- | --- | --- | --- | --- | --- |
|  |  | Case definition adequate | Representativeness of the cases | Selection of Controls | Definition of Controls | Comparability of cases and controls based on the design or analysis | Ascertainment of exposure | Same method of ascertainment for cases and controls | Non-Response rate |  |  |
| 21 | Zar 2016 | 🟉 | 🟉 | 🟉 | 🟉 | 🟉🟉 | 🟉 | 🟉 | 🟉 | 9 | LOW |
| 22 | Kelly 2017 | 🟉 | - | 🟉 | 🟉 | 🟉🟉 | 🟉 | 🟉 | - | 7 | LOW |
| 23 | Levine 2000 | 🟉 | - | - | 🟉 | 🟉🟉 | 🟉 | 🟉 | - | 6 | HIGH |
| 24 | Sutcliffe 2019 | 🟉 | - | 🟉 | 🟉 | 🟉 | 🟉 | 🟉 | 🟉 | 7 | LOW |
| 25 | Mogdasy 1992 | 🟉 | - | 🟉 | - | 🟉🟉 | 🟉 | 🟉 | - | 6 | HIGH |
| 26 | Macasaet 1968 | 🟉 | - | - | 🟉 | - | 🟉 | 🟉 | 🟉 | 5 | LOW |
| 27 | Bhuiyan 2019 | 🟉 | - | 🟉 | 🟉 | 🟉 | 🟉 | 🟉 | 🟉 | 7 | LOW |
| 28 | Liu 2005 | 🟉 | 🟉 | - | 🟉 | 🟉🟉 | 🟉 | 🟉 | 🟉 | 8 | LOW |
| 29 | Palmu 2019 | - | - | 🟉 | - | 🟉🟉 | 🟉 | 🟉 | - | 5 | HIGH |
| 30 | Anh 2007 | 🟉 | - | 🟉 | 🟉 | - | 🟉 | 🟉 | - | 5 | HIGH |
| 31 | Bhuyan 2017 | 🟉 | 🟉 | - | 🟉 | - | 🟉 | 🟉 | 🟉 | 6 | HIGH |
| 32 | Mastro 1993 | 🟉 | - | 🟉 | 🟉 | - | 🟉 | 🟉 | - | 5 | HIGH |
| 33 | Vathanophas 1990 | 🟉 | 🟉 | 🟉 | 🟉 | - | 🟉 | 🟉 | - | 6 | HIGH |
| 34 | Smith-Vaughan 2018 | 🟉 | - | 🟉 | 🟉 | 🟉🟉 | 🟉 | 🟉 | - | 7 | LOW |
| 35 | Coles 2009 | - | 🟉 | 🟉 | 🟉 | 🟉🟉 | 🟉 | 🟉 | 🟉 | 8 | LOW |
| 36 | Vu 2011 | 🟉 | 🟉 | 🟉 | 🟉 | 🟉🟉 | 🟉 | 🟉 | - | 8 | LOW |
| 37 | Bezerra 2011 | 🟉 | 🟉 | 🟉 | 🟉 | - | 🟉 | 🟉 | - | 6 | HIGH |
| 38 | Suarez-Arrabal 2015 | 🟉 | 🟉 | 🟉 | 🟉 | 🟉 | 🟉 | 🟉 | - | 7 | LOW |
| 39 | Kumar 2005 | 🟉 | - | 🟉 | 🟉 | 🟉 | 🟉 | 🟉 | 🟉 | 7 | LOW |

**Appendix 2**

**Quality assessment for cross-sectional and prospective case-control studies (continued)**

| Study number | Study details | Selection | | | | Comparability | Outcome | | | Score | Risk of bias |
| --- | --- | --- | --- | --- | --- | --- | --- | --- | --- | --- | --- |
|  |  | Case definition adequate | Representativeness of the cases | Selection of Controls | Definition of Controls | Comparability of cases and controls based on the design or analysis | Ascertainment of exposure | Same method of ascertainment for cases and controls | Non-Response rate |  |  |
| 40 | Kahane 1998 | 🟉 | 🟉 | - | 🟉 | - | 🟉 | 🟉 | - | 5 | HIGH |
| 41 | Man 2019 | 🟉 | - | 🟉 | 🟉 | 🟉🟉 | 🟉 | 🟉 | 🟉 | 8 | LOW |
| 42 | Teo 2015 | - | 🟉 | 🟉 | 🟉 | 🟉🟉 | 🟉 | 🟉 | 🟉 | 8 | LOW |
| 43 | Teo 2018 | - | 🟉 | 🟉 | 🟉 | 🟉🟉 | 🟉 | 🟉 | - | 7 | LOW |
| 44 | De Steenhuijsen Piters 2016 | 🟉 | - | 🟉 | 🟉 | 🟉 | 🟉 | 🟉 | 🟉 | 7 | LOW |
| 45 | Hasegawa 2017 | 🟉 | 🟉 | 🟉 | 🟉 | 🟉🟉 | 🟉 | 🟉 | 🟉 | 9 | LOW |
| 46 | Ederveen 2018 | 🟉 | 🟉 | 🟉 | 🟉 | 🟉🟉 | 🟉 | 🟉 | - | 8 | LOW |
| 47 | Hu 2017 | 🟉 | - | 🟉 | 🟉 | 🟉🟉 | 🟉 | 🟉 | - | 7 | LOW |
| 48 | Salter 2017 | 🟉 | - | 🟉 | - | - | 🟉 | 🟉 | - | 4 | HIGH |
| 49 | Carrion 2019 | 🟉 | - | 🟉 | - | 🟉🟉 | 🟉 | 🟉 | 🟉 | 7 | LOW |
| 50 | Montgomery 1990 | 🟉 | 🟉 | 🟉 | 🟉 | 🟉 | 🟉 | 🟉 | 🟉 | 8 | LOW |

PERCH - Pneumonia Etiology Research for Child Health
