# Supplementary material for: The association between bacteria colonizing the upper respiratory tract and lower respiratory tract infection in young children: a systematic review and meta-analysis
Source: Clin Microbiol Infect. 2021 Sep;27(9):1262–70. doi: 10.1016/j.cmi.2021.05.034 (PMC8437050; doi:10.1016/j.cmi.2021.05.034)
Supplement: Multimedia component 3 [file mmc3.docx]

**Appendix 3**

**Meta-analyses on upper respiratory tract bacteria prevalence data generated by studies using culture or polymerase chain reaction**


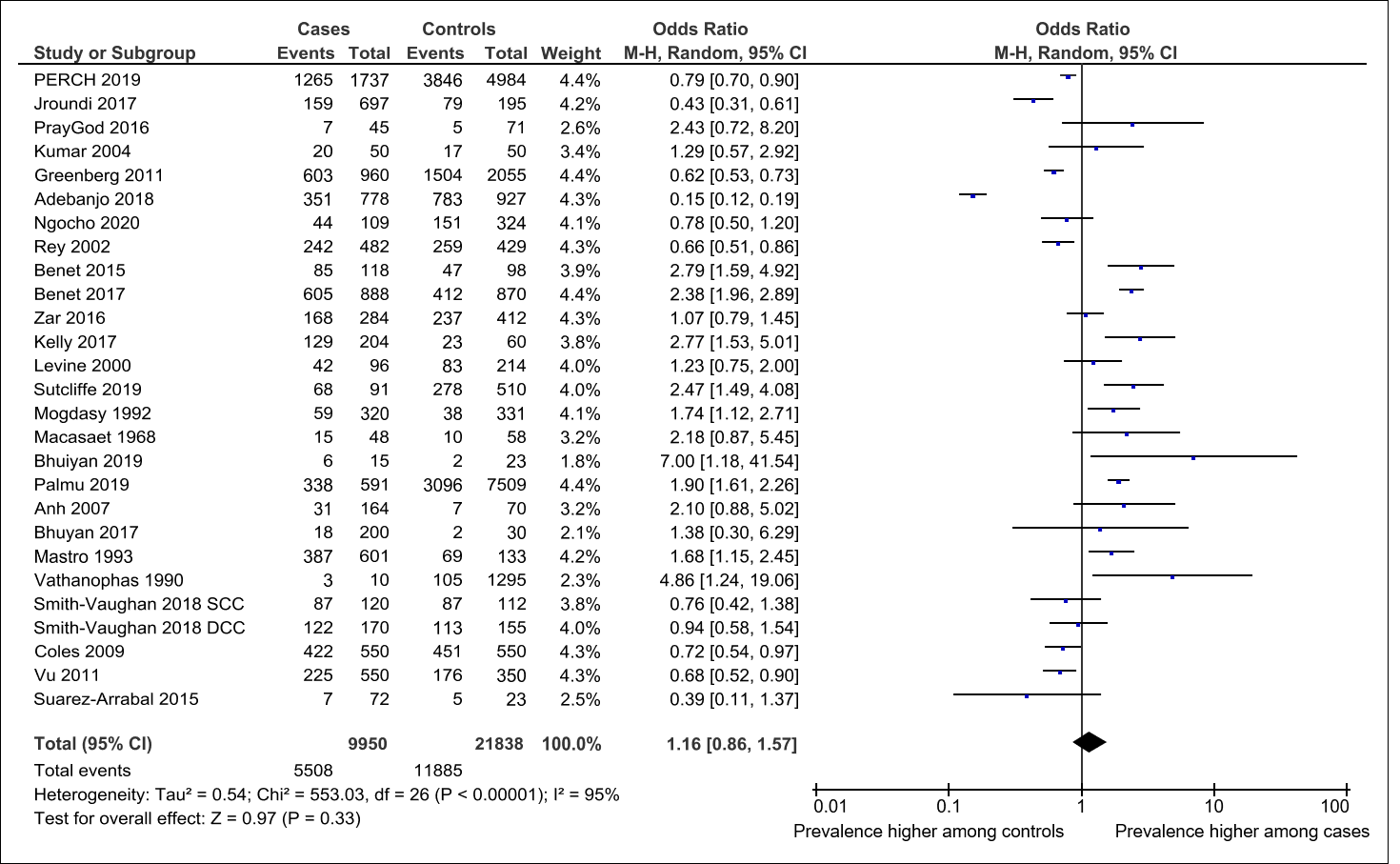


**Figure S1. Forest plot of the upper respiratory tract prevalence of *Streptococcus pneumoniae* from cases and controls**

Vertical line - “no difference point” between *S. pneumoniae* prevalence observed from cases and controls; squares - odds ratios; diamond - pooled odds ratio; horizontal lines - 95% confidence intervals. CI - confidence interval; DCC - Different Child Control cohort; M-H - Mantel-Haenszel; PERCH - Pneumonia Etiology Research for Child Health; SCC - Same Child Control cohort.

**
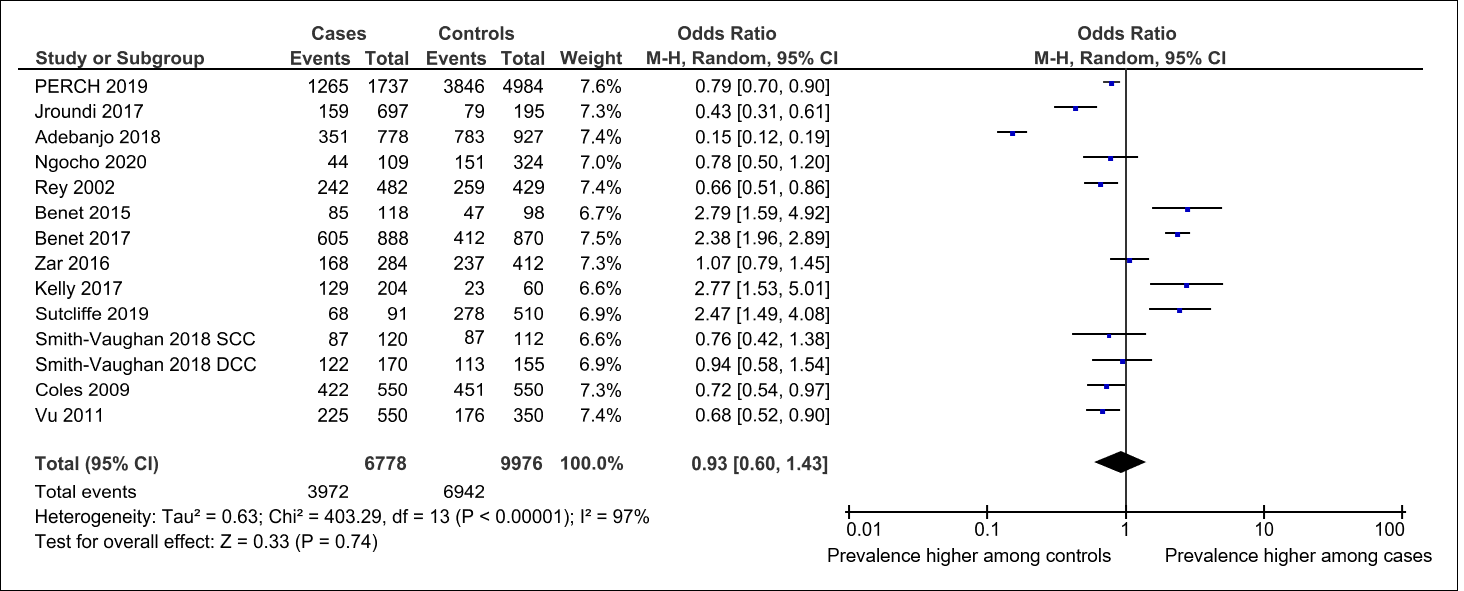
**

**Figure S2. Sensitivity analysis forest plot of the upper respiratory tract prevalence of *Streptococcus pneumoniae* from cases and controls**

Vertical line - “no difference point” between *S. pneumoniae* prevalence observed from cases and controls; squares - odds ratios; diamond - pooled odds ratio; horizontal lines - 95% confidence intervals. CI - confidence interval; DCC - Different Child Control cohort; M-H - Mantel-Haenszel; PERCH - Pneumonia Etiology Research for Child Health; SCC - Same Child Control cohort.

**Appendix 3**

**Meta-analyses on upper respiratory tract bacteria prevalence data generated by studies using culture or polymerase chain reaction (continued)**


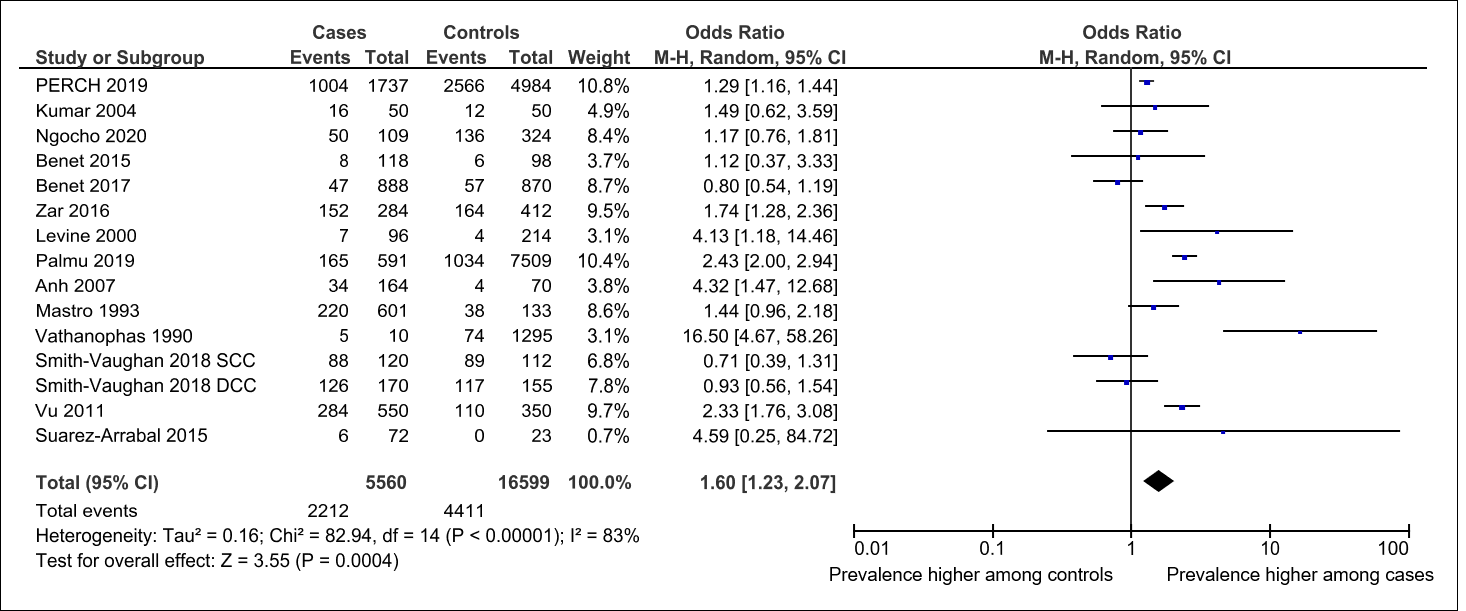


**Figure S3. Forest plot of the upper respiratory tract prevalence of *Haemophilus influenzae* from cases and controls**

Vertical line - “no difference point” between *H. influenzae* prevalence observed from cases and controls; squares - odds ratios; diamond - pooled odds ratio; horizontal lines - 95% confidence intervals. CI - confidence interval; DCC - Different Child Control cohort; M-H - Mantel-Haenszel; PERCH - Pneumonia Etiology Research for Child Health; SCC - Same Child Control cohort.

**
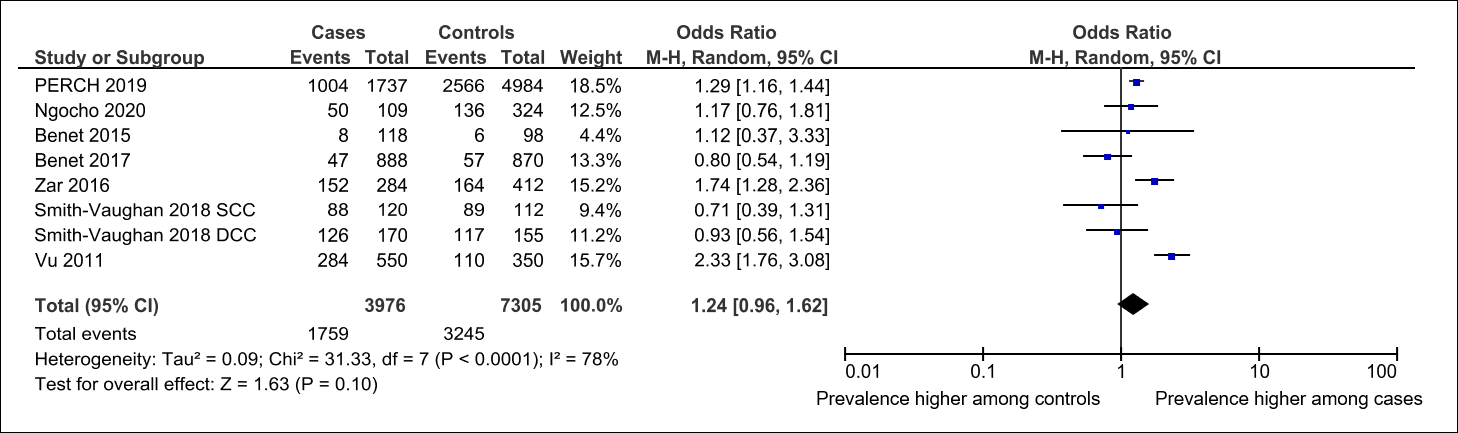
**

**Figure S4. Sensitivity analysis forest plot of the upper respiratory tract prevalence of *Haemophilus influenzae* from cases and controls**

Vertical line - “no difference point” between *H. influenzae* prevalence observed from cases and controls; squares - odds ratios; diamond - pooled odds ratio; horizontal lines - 95% confidence intervals. CI - confidence interval; DCC - Different Child Control cohort; M-H - Mantel-Haenszel; PERCH - Pneumonia Etiology Research for Child Health; SCC - Same Child Control cohort.

**Appendix 3**

**Meta-analyses on upper respiratory tract bacteria prevalence data generated by studies using culture or polymerase chain reaction (continued)**


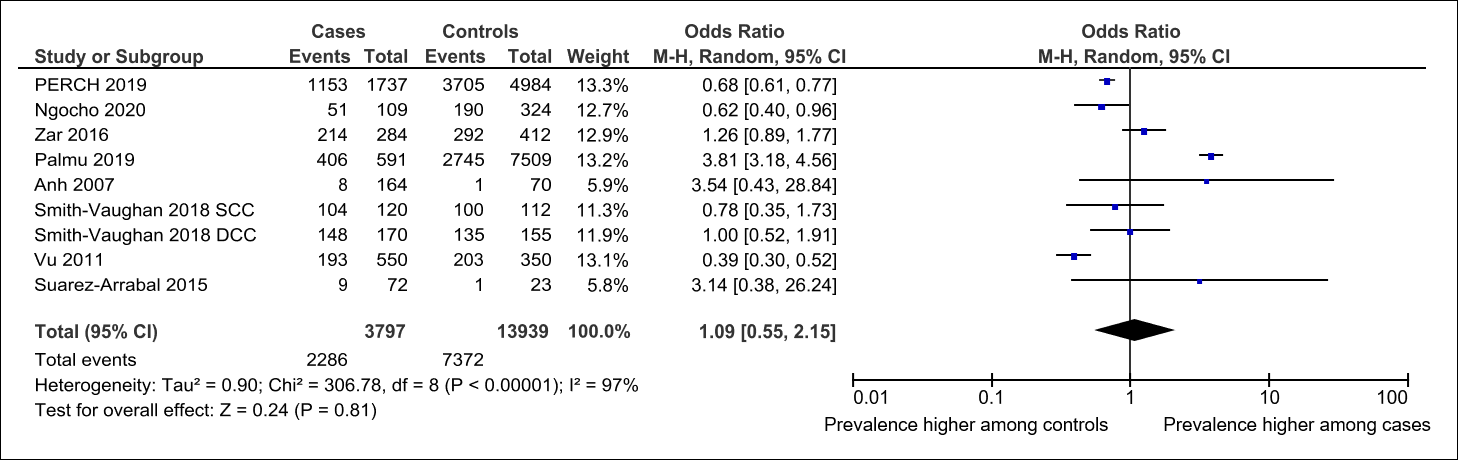


**Figure S5. Forest plot of the upper respiratory tract prevalence of *Moraxella catarrhalis* from cases and controls**

Vertical line - “no difference point” between *M. catarrhalis* prevalence observed from cases and controls; squares - odds ratios; diamond - pooled odds ratio; horizontal lines - 95% confidence intervals. CI - confidence interval; DCC - Different Child Control cohort; M-H - Mantel-Haenszel; PERCH - Pneumonia Etiology Research for Child Health; SCC - Same Child Control cohort.

**
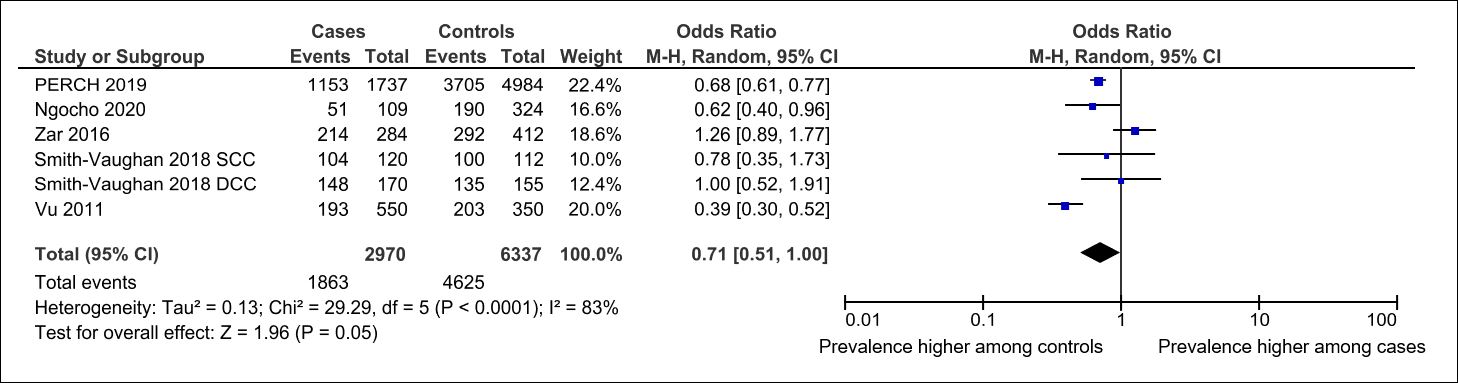
**

**Figure S6. Sensitivity analysis forest plot of the upper respiratory tract prevalence of *Moraxella catarrhalis* from cases and controls**

Vertical line - “no difference point” between *M. catarrhalis* prevalence observed from cases and controls; squares - odds ratios; diamond - pooled odds ratio; horizontal lines - 95% confidence intervals. CI - confidence interval; DCC - Different Child Control cohort; M-H - Mantel-Haenszel; PERCH - Pneumonia Etiology Research for Child Health; SCC - Same Child Control cohort.

**Appendix 3**

**Meta-analyses on upper respiratory tract bacteria prevalence data generated by studies using culture or polymerase chain reaction (continued)**


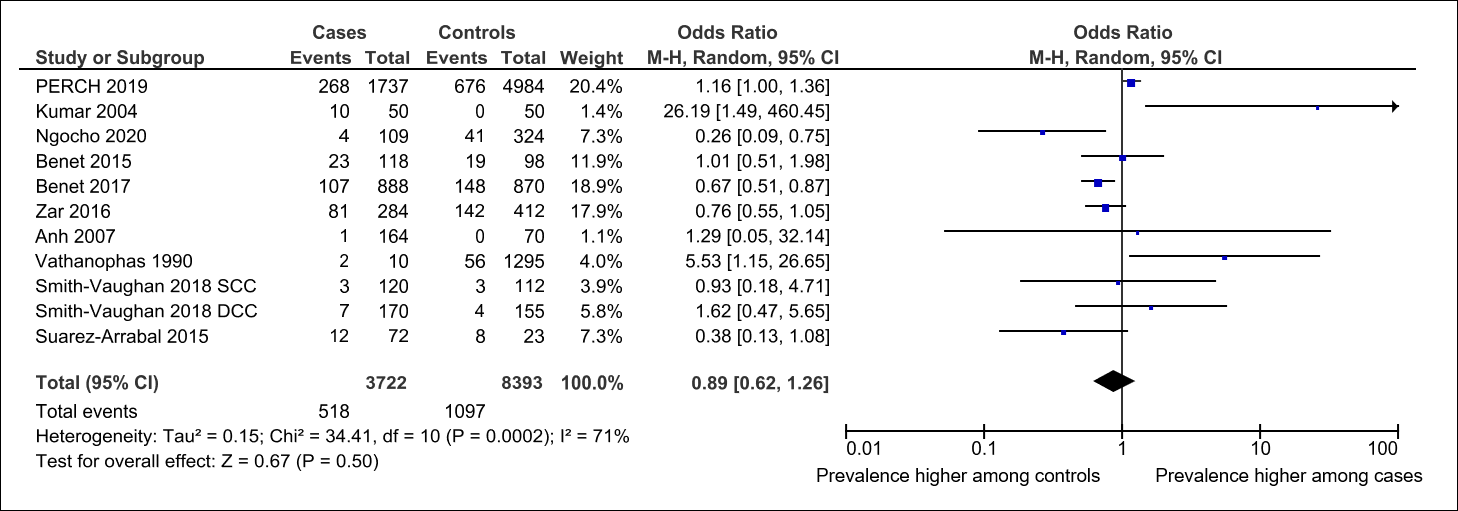


**Figure S7. Forest plot of the upper respiratory tract prevalence of *Staphylococcus aureus* from cases and controls**

Vertical line - “no difference point” between *S. aureus* prevalence observed from cases and controls; squares - odds ratios; diamond - pooled odds ratio; horizontal lines - 95% confidence intervals. CI - confidence interval; DCC - Different Child Control cohort; M-H - Mantel-Haenszel; PERCH - Pneumonia Etiology Research for Child Health; SCC - Same Child Control cohort.

**
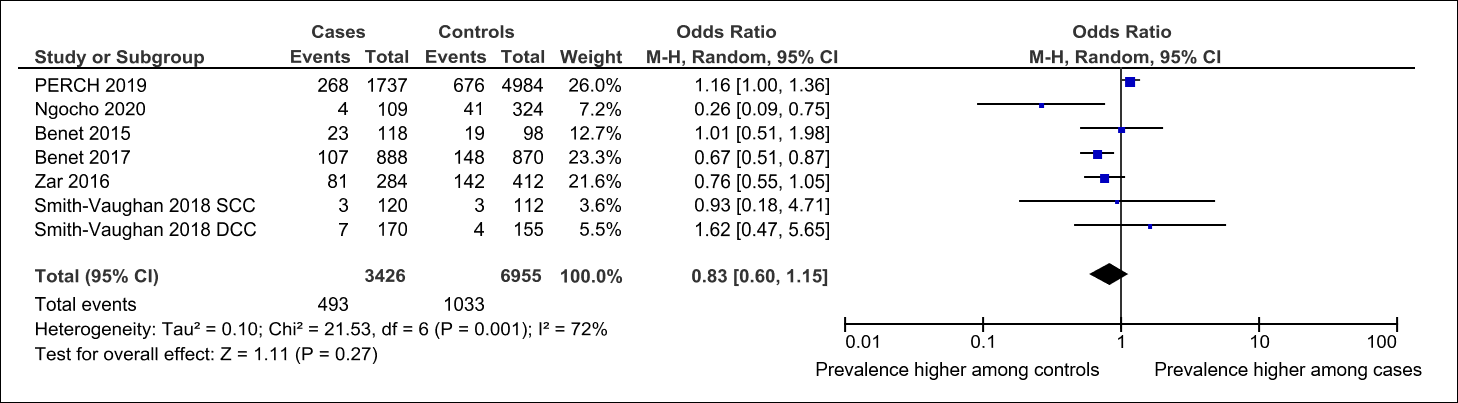
**

**Figure S8. Sensitivity analysis forest plot of the upper respiratory tract prevalence of *Staphylococcus aureus* from cases and controls**

Vertical line - “no difference point” between *S. aureus* prevalence observed from cases and controls; squares - odds ratios; diamond - pooled odds ratio; horizontal lines - 95% confidence intervals. CI - confidence interval; DCC - Different Child Control cohort; M-H - Mantel-Haenszel; PERCH - Pneumonia Etiology Research for Child Health; SCC - Same Child Control cohort.

**Appendix 3**

**Meta-analyses on upper respiratory tract bacteria prevalence data generated by studies using culture or polymerase chain reaction (continued)**


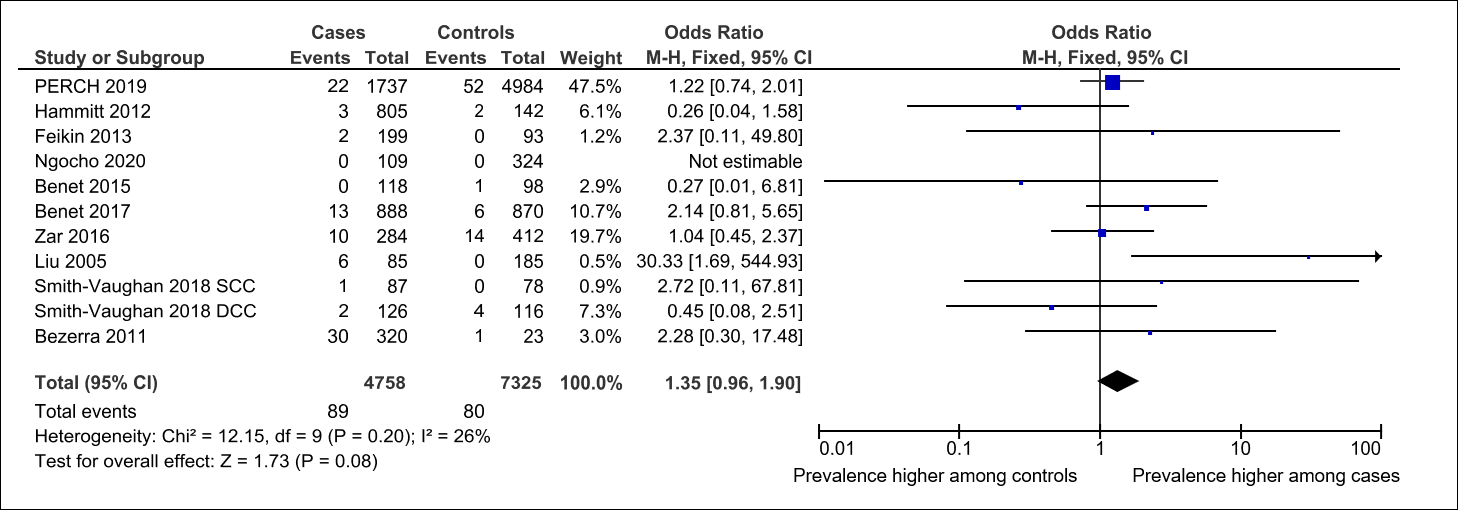


**Figure S9. Forest plot of the upper respiratory tract prevalence of *Mycoplasma pneumoniae* from cases and controls**

Vertical line - “no difference point” between *M. pneumoniae* prevalence observed from cases and controls; squares - odds ratios; diamond - pooled odds ratio; horizontal lines - 95% confidence intervals. CI - confidence interval; DCC - Different Child Control cohort; M-H - Mantel-Haenszel; PERCH - Pneumonia Etiology Research for Child Health; SCC - Same Child Control cohort.

**
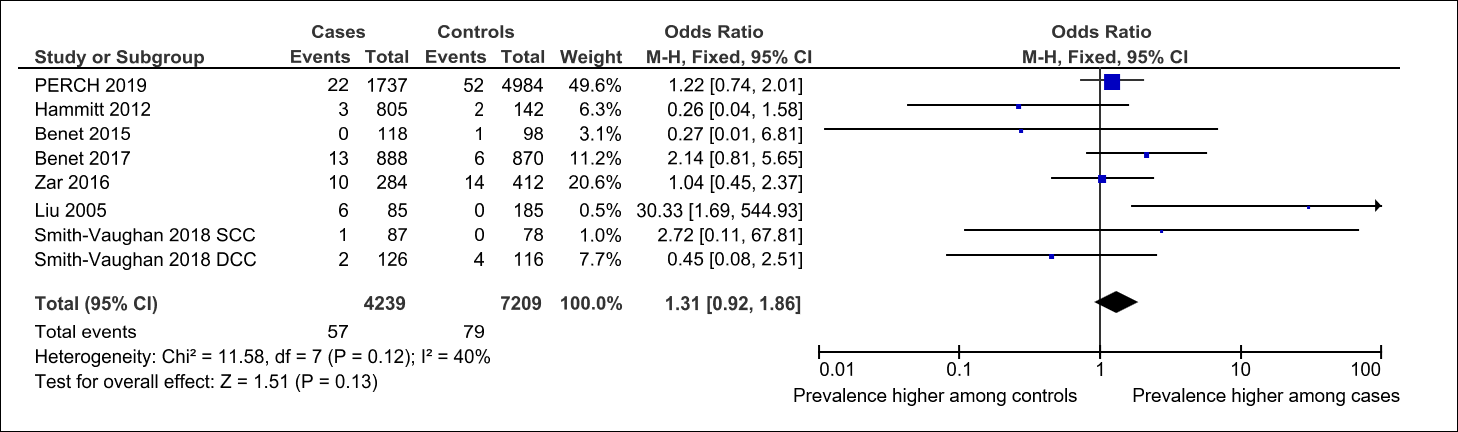
**

**Figure S10. Sensitivity analysis forest plot of the upper respiratory tract prevalence of *Mycoplasma pneumoniae* from cases and controls**

Vertical line - “no difference point” between *M. pneumoniae* prevalence observed from cases and controls; squares - odds ratios; diamond - pooled odds ratio; horizontal lines - 95% confidence intervals. CI - confidence interval; DCC - Different Child Control cohort; M-H - Mantel-Haenszel; PERCH - Pneumonia Etiology Research for Child Health; SCC - Same Child Control cohort.

**Appendix 3**

**Meta-analyses on upper respiratory tract bacteria prevalence data generated by studies using culture or polymerase chain reaction (continued)**


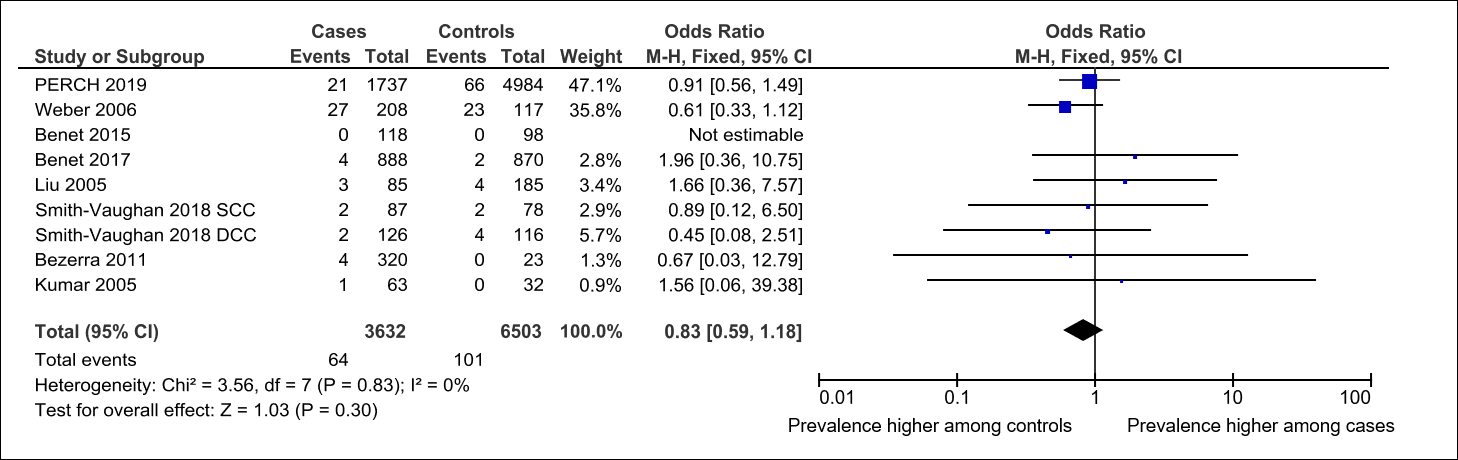


**Figure S11. Forest plot of the upper respiratory tract prevalence of *Chlamydophila pneumoniae* from cases and controls**

Vertical line - “no difference point” between *S. pneumoniae* prevalence observed from cases and controls; squares - odds ratios; diamond - pooled odds ratio; horizontal lines - 95% confidence intervals. CI - confidence interval; DCC - Different Child Control cohort; M-H - Mantel-Haenszel; PERCH - Pneumonia Etiology Research for Child Health; SCC - Same Child Control cohort.

**
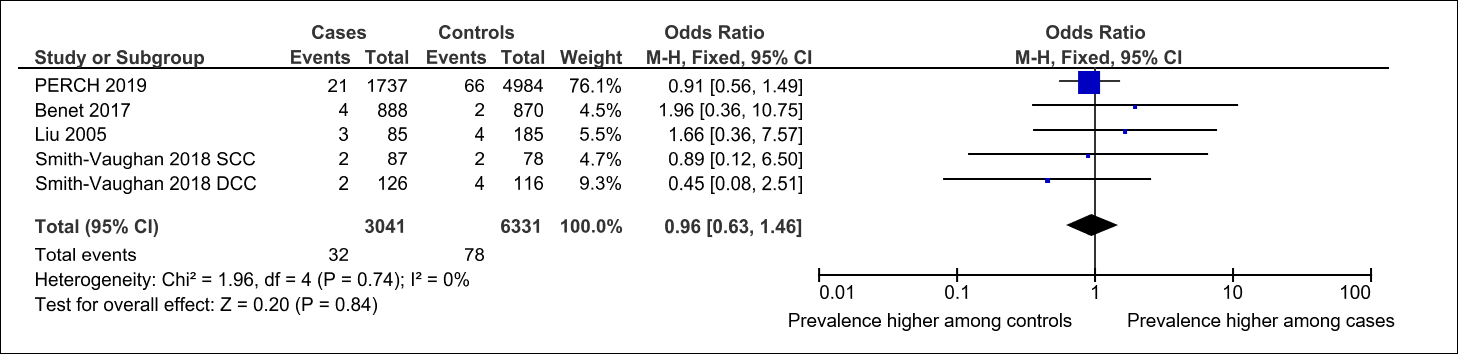
**

**Figure S12. Sensitivity analysis forest plot of the upper respiratory tract prevalence of *Streptococcus pneumoniae* from cases and controls**

Vertical line - “no difference point” between *S. pneumoniae* prevalence observed from cases and controls; squares - odds ratios; diamond - pooled odds ratio; horizontal lines - 95% confidence intervals. CI - confidence interval; DCC - Different Child Control cohort; M-H - Mantel-Haenszel; PERCH - Pneumonia Etiology Research for Child Health; SCC - Same Child Control cohort.

**Appendix 3**

**Meta-analyses on upper respiratory tract bacteria prevalence data generated by studies using culture or polymerase chain reaction (continued)**


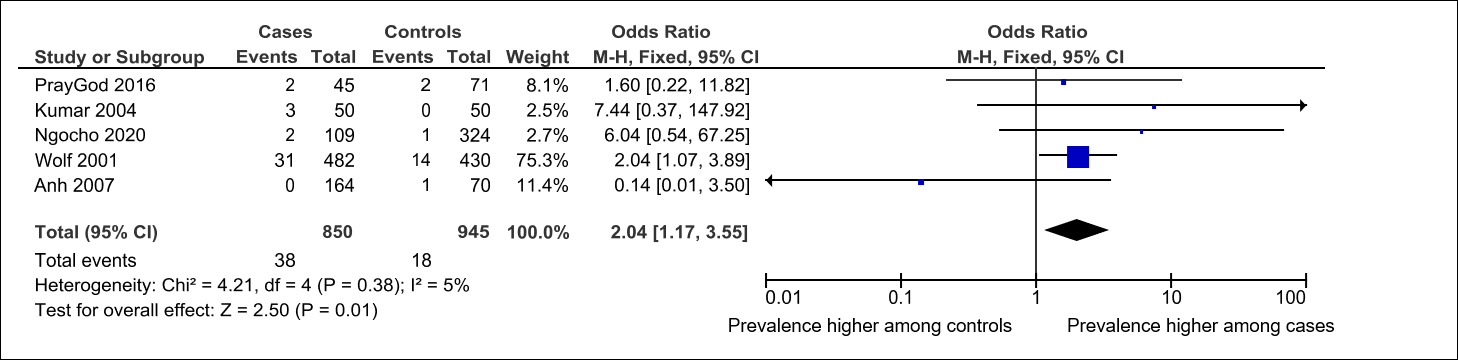


**Figure S13. Forest plot of the upper respiratory tract prevalence of *Klebsiella* spp. from cases and controls**

Vertical line - “no difference point” between *Klebsiella* spp. prevalence observed from cases and controls; squares - odds ratios; diamond - pooled odds ratio; horizontal lines - 95% confidence intervals. CI - confidence interval; M-H - Mantel-Haenszel.

**
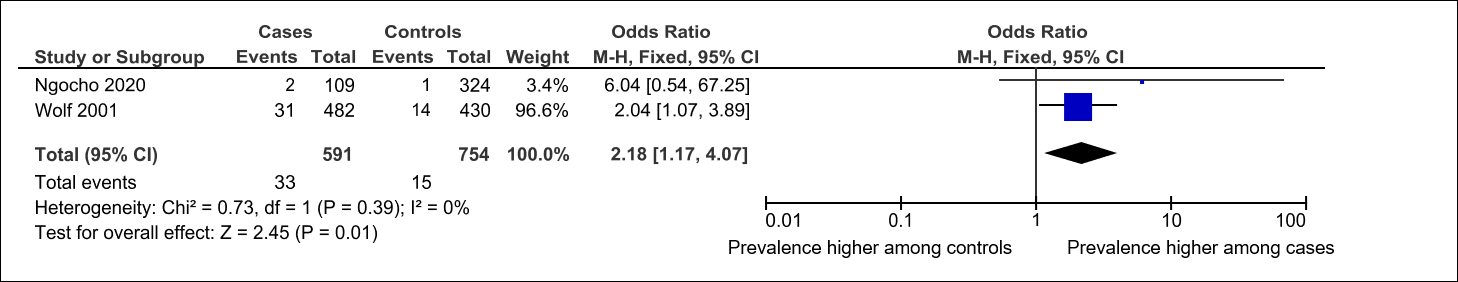
**

**Figure S14. Sensitivity analysis forest plot of the upper respiratory tract prevalence of *Klebsiella* spp. from cases and controls**

Vertical line - “no difference point” between *Klebsiella* spp. prevalence observed from cases and controls; squares - odds ratios; diamond - pooled odds ratio; horizontal lines - 95% confidence intervals. CI - confidence interval; M-H - Mantel-Haenszel.

**Appendix 3**

**Subgroup analyses**

**Table S1 – *Streptococcus pneumoniae* prevalence among cases and controls**

| Analysis category | Subgroup analysis | Number of studies included | Cases screened (N) | Controls screened (N) | Heterogeneity (I^2^) | Analysis method | Odds ratio | 95% confidence interval | p-value |
| --- | --- | --- | --- | --- | --- | --- | --- | --- | --- |
| Country income groups  (World Bank classification) | Low- and low-middle-income countries | 15 | 6862 | 9574 | 97% | Mantel-Haenszel, Random effects model | 1.07 | 0.68-1.68 | 0.77 |
|  | Upper-middle- and high-income countries | 11 | 3088 | 12264 | 92% | Mantel-Haenszel, Random effects model | 1.30 | 0.87-1.94 | 0.21 |
| Laboratory technique | Culture | 16 | 5164 | 6941 | 94% | Mantel-Haenszel, Random effects model | 1.05 | 0.67-1.63 | 0.84 |
|  | Polymerase chain reaction | 10 | 4786 | 14897 | 94% | Mantel-Haenszel, Random effects model | 1.33 | 0.93-1.90 | 0.12 |
| Participant age range | 0-24 months | 6 | 1456 | 8294 | 81% | Mantel-Haenszel, Random effects model | 1.31 | 0.86-1.99 | 0.21 |
|  | 0-60 months | 20 | 8494 | 13544 | 96% | Mantel-Haenszel, Random effects model | 1.12 | 0.79-1.59 | 0.54 |
| Case definition | Severe or very severe pneumonia | 4 | 2529 | 5300 | 82% | Mantel-Haenszel, Random effects model | 0.82 | 0.49-1.36 | 0.44 |
|  | Pneumonia | 13 | 4393 | 6311 | 97% | Mantel-Haenszel, Random effects model | 1.29 | 0.74-2.25 | 0.36 |
|  | Lower respiratory tract infection | 8 | 2956 | 10204 | 88% | Mantel-Haenszel, Random effects model | 1.22 | 0.81-1.83 | 0.34 |
| Antibiotic administration prior to sampling | Higher proportion of cases had antibiotics administered compared to controls | 8 | 2420 | 7230 | 97% | Mantel-Haenszel, Random effects model | 0.75 | 0.43-1.31 | 0.32 |

**Appendix 3**

**Subgroup analyses (continued)**

**Table S2 – *Haemophilus influenzae* prevalence among cases and controls**

| Analysis category | Subgroup analysis | Number of studies included | Cases screened (N) | Controls screened (N) | Heterogeneity (I^2^) | Analysis method | Odds ratio | 95% confidence interval | p-value |
| --- | --- | --- | --- | --- | --- | --- | --- | --- | --- |
| Country income groups  (World Bank classification) | Low- and low-middle-income countries | 9 | 4501 | 7291 | 73% | Mantel-Haenszel, Random effects model | 1.47 | 1.15-1.87 | 0.002 |
|  | Upper-middle- and high-income countries | 5 | 1059 | 9308 | 86% | Mantel-Haenszel, Random effects model | 2.24 | 1.06-4.72 | 0.03 |
| Laboratory technique | Culture | 6 | 993 | 1785 | 71% | Mantel-Haenszel, Random effects model | 3.26 | 1.50-7.11 | 0.003 |
|  | Polymerase chain reaction | 8 | 4567 | 14814 | 87% | Mantel-Haenszel, Random effects model | 1.35 | 1.02-1.79 | 0.04 |
| Participant age range | 0-24 months | 4 | 1237 | 8211 | 84% | Mantel-Haenszel, Random effects model | 1.42 | 0.87-2.33 | 0.16 |
|  | 0-60 months | 20 | 4323 | 8388 | 80% | Mantel-Haenszel, Random effects model | 1.71 | 1.23-2.36 | 0.001 |
| Case definition | Severe or very severe pneumonia | 2 | 1787 | 5034 | 0% | Mantel-Haenszel, Fixed effects model | 1.29 | 1.16-1.44 | <0.00001 |
|  | Pneumonia | 5 | 1495 | 1918 | 69% | Mantel-Haenszel, Random effects model | 1.32 | 0.86-2.04 | 0.21 |
|  | Lower respiratory tract infection | 6 | 2206 | 9624 | 85% | Mantel-Haenszel, Random effects model | 1.94 | 1.26-2.98 | 0.003 |
| Antibiotic administration prior to sampling | Higher proportion of cases had antibiotics administered compared to controls | 3 | 2383 | 5548 | 89% | Mantel-Haenszel, Random effects model | 1.93 | 1.11-3.37 | 0.02 |

**Appendix 3**

**Subgroup analyses (continued)**

**Table S3 – *Moraxella catarrhalis* prevalence among cases and controls**

| Analysis category | Subgroup analysis | Number of studies included | Cases screened (N) | Controls screened (N) | Heterogeneity (I^2^) | Analysis method | Odds ratio | 95% confidence interval | p-value |
| --- | --- | --- | --- | --- | --- | --- | --- | --- | --- |
| Country income groups  (World Bank classification) | Low- and low-middle-income countries | 5 | 2844 | 6140 | 87% | Mantel-Haenszel, Random effects model | 0.71 | 0.47-1.07 | 0.10 |
|  | Upper-middle- and high-income countries | 3 | 953 | 7799 | 89% | Mantel-Haenszel, Random effects model | 1.66 | 0.60-4.63 | 0.33 |
| Laboratory technique | Culture | 2 | 236 | 93 | 0% | Mantel-Haenszel, Fixed effects model | 3.34 | 0.75-14.85 | 0.11 |
|  | Polymerase chain reaction | 6 | 2269 | 13846 | 98% | Mantel-Haenszel, Random effects model | 0.94 | 0.45-1.94 | 0.86 |
| Participant age range | 0-24 months | 4 | 1237 | 8211 | 92% | Mantel-Haenszel, Random effects model | 1.56 | 0.71-3.40 | 0.27 |
|  | 0-60 months | 4 | 2560 | 5728 | 81% | Mantel-Haenszel, Random effects model | 0.59 | 0.39-0.87 | 0.008 |
| Case definition | Severe or very severe pneumonia | 1 | - | - | - | - | - | - | - |
|  | Pneumonia | 2 | 393 | 736 | 84% | Mantel-Haenszel, Random effects model | 0.89 | 0.45-1.79 | 0.75 |
|  | Lower respiratory tract infection | 4 | 1595 | 8196 | 98% | Mantel-Haenszel, Random effects model | 1.24 | 0.34-4.57 | 0.74 |
| Antibiotic administration prior to sampling | Higher proportion of cases had antibiotics administered compared to controls | 2 | 2287 | 5334 | 92% | Mantel-Haenszel, Random effects model | 0.52 | 0.30-0.90 | 0.02 |

**Appendix 3**

**Subgroup analyses (continued)**

**Table S4 – *Staphylococcus aureus* prevalence among cases and controls**

| Analysis category | Subgroup analysis | Number of studies included | Cases screened (N) | Controls screened (N) | Heterogeneity (I^2^) | Analysis method | Odds ratio | 95% confidence interval | p-value |
| --- | --- | --- | --- | --- | --- | --- | --- | --- | --- |
| Country income groups  (World Bank classification) | Low- and low-middle-income countries | 7 | 3350 | 6808 | 77% | Mantel-Haenszel, Random effects model | 0.83 | 0.57-1.22 | 0.35 |
|  | Upper-middle- and high-income countries | 3 | 372 | 1585 | 65% | Mantel-Haenszel, Random effects model | 1.23 | 0.40-3.84 | 0.72 |
| Laboratory technique | Culture | 4 | 296 | 1438 | 77% | Mantel-Haenszel, Random effects model | 2.46 | 0.32-18.81 | 0.38 |
|  | Polymerase chain reaction | 6 | 3426 | 6955 | 72% | Mantel-Haenszel, Random effects model | 0.83 | 0.60-1.15 | 0.27 |
| Participant age range | 0-24 months | 3 | 646 | 702 | 6% | Mantel-Haenszel, Fixed effects model | 0.76 | 0.56-1.02 | 0.07 |
|  | 0-60 months | 7 | 3076 | 7691 | 79% | Mantel-Haenszel, Random effects model | 0.99 | 0.60-1.62 | 0.96 |
| Case definition | Severe or very severe pneumonia | 2 | 1787 | 5034 | 78% | Mantel-Haenszel, Random effects model | 3.94 | 0.20-79.06 | 0.37 |
|  | Pneumonia | 4 | 1399 | 1704 | 38% | Mantel-Haenszel, Fixed effects model | 0.69 | 0.57-0.84 | 0.0002 |
|  | Lower respiratory tract infection | 3 | 464 | 1632 | 0% | Mantel-Haenszel, Fixed effects model | 1.67 | 0.72-3.87 | 0.24 |
| Antibiotic administration prior to sampling | Higher proportion of cases had antibiotics administered compared to controls | 1 | - | - | - | - | - | - | - |

**Appendix 3**

**Subgroup analyses (continued)**

**Table S5 – *Mycoplasma pneumoniae* prevalence among cases and controls**

| Analysis category | Subgroup analysis | Number of studies included | Cases screened (N) | Controls screened (N) | Heterogeneity (I^2^) | Analysis method | Odds ratio | 95% confidence interval | p-value |
| --- | --- | --- | --- | --- | --- | --- | --- | --- | --- |
| Country income groups  (World Bank classification) | Low- and low-middle-income countries | 6 | 4140 | 6923 | 5% | Mantel-Haenszel, Fixed effects model | 1.21 | 0.83-1.75 | 0.32 |
|  | Upper-middle- and high-income countries | 3 | 618 | 402 | 55% | Mantel-Haenszel, Random effects model | 2.38 | 0.41-13.80 | 0.33 |
| Laboratory technique | Culture | 0 | - | - | - | - | - | - | - |
|  | Polymerase chain reaction | 9 | - | - | - | - | - | - | - |
| Participant age range | 0-24 months | 2 | 497 | 606 | 0% | Mantel-Haenszel, Fixed effects model | 0.94 | 0.46-1.92 | 0.86 |
|  | 0-60 months | 7 | 4261 | 6719 | 42% | Mantel-Haenszel, Random effects model | 1.44 | 0.67-3.11 | 0.35 |
| Case definition | Severe or very severe pneumonia | 3 | 2741 | 5219 | 30% | Mantel-Haenszel, Fixed effects model | 1.14 | 0.70-1.84 | 0.60 |
|  | Pneumonia | 4 | 1484 | 1889 | 56% | Mantel-Haenszel, Random effects model | 1.78 | 0.57-5.49 | 0.32 |
|  | Lower respiratory tract infection | 2 | 533 | 217 | 0% | Mantel-Haenszel, Fixed effects model | 1.13 | 0.38-3.33 | 0.83 |
| Antibiotic administration prior to sampling | Higher proportion of cases had antibiotics administered compared to controls | 2 | 1822 | 5169 | 80% | Mantel-Haenszel, Random effects model | 4.47 | 0.18-108.65 | 0.36 |

**Appendix 3**

**Subgroup analyses (continued)**

**Table S6 – *Chlamydophila pneumoniae* prevalence among cases and controls**

| Analysis category | Subgroup analysis | Number of studies included | Cases screened (N) | Controls screened (N) | Heterogeneity (I^2^) | Analysis method | Odds ratio | 95% confidence interval | p-value |
| --- | --- | --- | --- | --- | --- | --- | --- | --- | --- |
| Country income groups  (World Bank classification) | Low- and low-middle-income countries | 3 | 2951 | 6069 | 5% | Mantel-Haenszel, Fixed effects model | 0.82 | 0.56-1.19 | 0.30 |
|  | Upper-middle- and high-income countries | 4 | 681 | 434 | 0% | Mantel-Haenszel, Fixed effects model | 0.92 | 0.38-2.24 | 0.85 |
| Laboratory technique | Culture | 0 | - | - | - | - | - | - | - |
|  | Polymerase chain reaction | 7 | - | - | - | - | - | - | - |
| Participant age range | 0-24 months | 2 | 276 | 226 | 0% | Mantel-Haenszel, Fixed effects model | 0.69 | 0.22-2.22 | 0.53 |
|  | 0-60 months | 5 | 3356 | 6277 | 0% | Mantel-Haenszel, Fixed effects model | 0.85 | 0.59-1.22 | 0.38 |
| Case definition | Severe or very severe pneumonia | 1 | - | - | - | - | - | - | - |
|  | Pneumonia | 3 | 1299 | 1270 | 26% | Mantel-Haenszel, Fixed effects model | 0.78 | 0.46-1.34 | 0.37 |
|  | Lower respiratory tract infection | 2 | 533 | 217 | 0% | Mantel-Haenszel, Fixed effects model | 0.61 | 0.19-1.97 | 0.41 |
| Antibiotic administration prior to sampling | Higher proportion of cases had antibiotics administered compared to controls | 2 | 1822 | 5169 | 0% | Mantel-Haenszel, Fixed effects model | 0.96 | 0.60-1.54 | 0.87 |

**Appendix 3**

**Subgroup analyses (continued)**

**Table S7 – *Klebsiella* spp. prevalence among cases and controls**

| Analysis category | Subgroup analysis | Number of studies included | Cases screened (N) | Controls screened (N) | Heterogeneity (I^2^) | Analysis method | Odds ratio | 95% confidence interval | p-value |
| --- | --- | --- | --- | --- | --- | --- | --- | --- | --- |
| Country income groups  (World Bank classification) | Low- and low-middle-income countries | 4 | 368 | 515 | 29% | Mantel-Haenszel, Fixed effects model | 2.01 | 0.66-6.10 | 0.22 |
|  | Upper-middle- and high-income countries | 1 | - | - | - | - | - | - | - |
| Laboratory technique | Culture | 4 | 741 | 621 | 12% | Mantel-Haenszel, Fixed effects model | 1.92 | 1.08-3.41 | 0.03 |
|  | Polymerase chain reaction | 1 | - | - | - | - | - | - | - |
| Participant age range | 0-24 months | 0 | - | - | - | - | - | - | - |
|  | 0-60 months | 5 | - | - | - | - | - | - | - |
| Case definition | Severe or very severe pneumonia | 2 | 95 | 121 | 0% | Mantel-Haenszel, Fixed effects model | 3.00 | 0.63-14.23 | 0.17 |
|  | Pneumonia | 2 | 591 | 754 | 0% | Mantel-Haenszel, Fixed effects model | 2.18 | 1.17-4.07 | 0.01 |
|  | Lower respiratory tract infection | 1 | - | - | - | - | - | - | - |
| Antibiotic administration prior to sampling | Higher proportion of cases had antibiotics administered compared to controls | 1 | - | - | - | - | - | - | - |
