# Supplementary material for: The association between bacteria colonizing the upper respiratory tract and lower respiratory tract infection in young children: a systematic review and meta-analysis
Source: Clin Microbiol Infect. 2021 Sep;27(9):1262–70. doi: 10.1016/j.cmi.2021.05.034 (PMC8437050; doi:10.1016/j.cmi.2021.05.034)
Supplement: Multimedia component 4 [file mmc4.docx]

**Appendix 4**

***Streptococcus pneumoniae* serotype data**

| Study number | Study details | *S. pneumoniae* prevalence | Vaccine formulation used for serotype classification | Other serotype classification employed | Serotyping results | | |
| --- | --- | --- | --- | --- | --- | --- | --- |
|  |  |  |  |  | Serotypes/-groups | Prevalence | Significance tested |
| 1 | PERCH Study Group 2019 | ⭡ among controls | PCV-13 | - | Vaccine types* | ⭡ among cases^1^ | yes^1^ |
|  |  |  |  |  | Non-vaccine types* | ⭡ among cases^1^ | yes^1^ |
| 7 | Jroundi 2016 | ⭡ among controls | PCV-13 | - | Typeable pneumococci: 3, 6A/B, 7FA, 9V/A/N/L, 18C/B, 19A/F/B/C, 23F | ⭡ among cases | not reported |
|  |  |  |  |  | Non-typable pneumococci  Typeable pneumococci: 4, 8^2^, 23A^2^ | ⭡ among controls | not reported |
| 12 | Greenberg 2011 | ⭡ among controls | PCV-7  PCV-10  PCV-13 | - | 1*, 5*, 7F*, 9V*, 14*, 19A*, 22F*^2^ | ⭡ among cases | yes |
|  |  |  |  |  | 6A*/B*, 23A*^2^, 35B*^2^ | ⭡ among controls |  |
| 13 | Adebanjo 2018 | ⭡ among controls | PCV-10 | - | Vaccine types*: 1, 4, 5, 6B, 7F, 9V, 14, 18C, 19F, 23F | ⭡ among cases | yes |
| 15 | Rey 2002 | ⭡ among controls | - | Paediatric serogroups | 6*, 14*, 19*, 23* | ⭡ among cases | yes |
| 18 | Bénet 2015 | ⭡ among cases | - | - | 1*, 5*, 6A/B | ⭡ among cases | yes |
| 24 | Sutcliffe 2019 | ⭡ among cases | PCV-13 | - | Vaccine types,  9N^2^, 10A*^2^, 11A^2^, 14, 15B^2^, 16F*^2^, 17F^2^, 19A*/F, 23B^2^/F*, 31^2^, 33B*^2^, 34*^2^, 35A*^2^/35B^2^ | ⭡ among cases | yes |
|  |  |  |  |  | 4, 5, 6A/B/C^2^, 8^2^, 9V, 10B^2^/F^2^, 13^2^, 15C^2^, 18C, 20^2^, 21^2^, 22A^2^, 23A^2^, 27^2^, 33F^2^ | ⭡ among controls | yes |
| 32 | Mastro 1993 | ⭡ among cases | PPSV23 | Paediatric vaccine type serogroups  Non-paediatric vaccine type serogroups  Non-vaccine types | Vaccine types: 1-5, 6, 7, 12, 14-15, 17-20, 22, 23 | ⭡ among cases | not reported |
|  |  |  |  |  | Non-vaccine types | ⭡ among controls | not reported |

**Appendix 4**

***Streptococcus pneumoniae* serotype data (continued)**

| Study number | Study details | *S. pneumoniae* prevalence | Vaccine formulation used for serotype classification | Other serotype classification employed | Serotyping results | | |
| --- | --- | --- | --- | --- | --- | --- | --- |
| 35 | Coles 2009 | ⭡ among controls | PCV-7  PCV-10  PCV-13 | - | 8^2^, 9, 10^2^, 15^2^, 17^2^, 19, 35^2^ | ⭡ among cases | yes |
|  |  |  |  |  | 3, 6, 14, 23 | ⭡ among controls | yes |
|  |  |  |  |  | Non-vaccine types | ⭡ among controls | yes |
| 36 | Vu 2011 | ⭡ among controls | PCV-13 | - | Vaccine types: 19F* | ⭡ among cases | yes |
| 48 | Salter 2017 |  |  |  | Non-typable, 1-3, 6A/B/C, 7B, 8, 9N, 10A/B/F, 11A, 12F, 13-14, 15A/B/C, 16F, 17F, 18C/F, 19A/B/F, 20-21, 22F, 23A/F, 24A, 28F, 33B/C, 34, 35C, 37-38, 45 | acquisition of a new serotype does not usually coincide with disease or a recognisable shift in proportional abundance of *S. pneumoniae* | not reported |
| 50 | Montgomery 1990 |  |  | Invasive (class I)  Moderately invasive (class II)  Rarely invasive (class III) | Invasive (class I) serotypes*: 1-5, 7, 9-10, 14, 18, 45-46 | ⭡ among cases | yes |
|  |  |  |  |  | Rarely invasive (class III) serotypes*: 13, 15-17, 20-22, 24-25, 28-29, 31, 33-36, 38-39, 42-43, 47 | ⭡ among controls | yes |

* significant p-values / adjusted Odds Ratios

^1.^ Subset of participants (235 cases and 380 controls) with high density *S. pneumoniae* on PCR

^2.^ Serotypes not included in pneumococcal conjugate vaccines (PCVs) or pneumococcal polysaccharide vaccine (PPSV23)
