# Supplementary material for: The association between bacteria colonizing the upper respiratory tract and lower respiratory tract infection in young children: a systematic review and meta-analysis
Source: Clin Microbiol Infect. 2021 Sep;27(9):1262–70. doi: 10.1016/j.cmi.2021.05.034 (PMC8437050; doi:10.1016/j.cmi.2021.05.034)
Supplement: Multimedia component 5 [file mmc5.docx]

**Appendix 5**

**Bacterial-bacterial URT co-detection from cases and controls**

| Study number | Study details | Co-detection description | Cases | | Controls | | p-value |
| --- | --- | --- | --- | --- | --- | --- | --- |
|  |  |  | n/N | % | n/N | % |  |
| 1 | PERCH Study Group 2019 | Two or more bacteria | 113/1737 | 7 | 736/4984 | 15 | <0.0001 |
|  |  | Two or more bacteria (with NP/OP PCR thresholds applied for SP and HI) | 82/1737 | 5 | 407/4984 | 8 | <0.0001 |
| 14 | Ngocho 2020 | SP + HI | 31/109 | 28 | 91/324 | 28 | 0.032 |
|  |  | SP + HI + MC | 20/109 | 18 | 82/324 | 25 | 0.002 |
| 16 | Wolf 2001 | Co-habitant Gram negative bacteria (particularly *Acinetobacter* and *Pseudomonas*) | 20/482 | 4 | 3/430 | 1 | <0.01 |
| 21 | Zar 2016 | 21 bacterial-bacterial co-occurrence patterns | Refer to study |  | Refer to study |  | NS |
| 29 | Palmu 2019 | Two or more bacteria including SP, HI and MC | 323/591 | 55 | 2086/7509^1^ | 28^1^ | NR |
| 32 | Mastro 1993 | SP + HI | 165/601 | 28 | 31/133 | 23 | NS |
| 33 | Vathanophas 1990 | SP + HI | 5/10 | 50 | 29/1295 | 2 | NR |
|  |  | SP + HI + MC | 2/10 | 20 | 3/1295 | 0.2 | NR |
| 34 | Smith-Vaughan 2018^2^ | SP + HI | 67/119 | 56 | 70/112 | 63 | NS |
|  |  | SP + MC | 78/119 | 66 | 80/112 | 72 | NS |
|  |  | HI + MC | 82/119 | 69 | 83/112 | 74 | NS |
| 36 | Vu 2011 | SP + HI | 61/550^3^ | 11^3^ | 15/350 | 4 | <0.0001^4^  0.04^5^ |
|  |  | SP + MC | 30/550^3^ | 6^3^ | 54/350 | 15 | <0.0001^4^  0.002^5^ |
|  |  | HI + MC | 59/550^3^ | 11^3^ | 17/350 | 5 | 0.36^4^  <0.0001^5^ |
|  |  | SP + HI + MC | 66/550^3^ | 12^3^ | 66/350 | 19 | <0.0001^4^  0.28^5^ |
| 38 | Suarez-Arrabal 2015 | Growth of more than one bacterium | 23/72 | 32 | 1/23 | 4 | 0.02 |

^1.^All specimens excluding specimens collected during lower respiratory tract infection (LRTI)

^2.^Co-detection data reported from same child cohort

^3.^Results from “radiologically confirmed pneumonia” and “other LRTI” cases combined

^4.^Comparison between “radiologically confirmed pneumonia” cases and controls

^5.^Comparison between “other LRTI” cases and controls

HI - *Haemophilus influenzae*; MC - *Moraxella catarrhalis*; NR - Not reported; NS - Not significant; PERCH - Pneumonia Etiology Research for Child Health; SP - *Streptococcus pneumoniae*
